# Competition and synergy of Arp2/3 and formins in nucleating actin waves

**DOI:** 10.1016/j.celrep.2024.114423

**Published:** 2024-07-04

**Authors:** Xiang Le Chua, Chee San Tong, Maohan Su, X.J. Xǔ, Shengping Xiao, Xudong Wu, Min Wu

**Affiliations:** 1 Department of Cell Biology, Yale University School of Medicine, New Haven, CT 06510, USA; 2 Department of Biological Sciences, Centre for Bioimaging Sciences, Singapore 117557, Singapore; 3 Mechanobiology Institute, National University of Singapore, Singapore 117411, Singapore; 4 Department of Physics, Yale University, New Haven, CT 06511, USA; 5 School of Life Sciences, Westlake University, Hangzhou 310024, China; 6 Present address: Faculty of Biological and Environmental Sciences & HiLIFE Institute of Biotechnology, University of Helsinki, FI-00014, Helsinki, Finland; 7 Lead contact

## Abstract

Actin assembly and dynamics are crucial for maintaining cell structure and changing physiological states. The broad impact of actin on various cellular processes makes it challenging to dissect the specific role of actin regulatory proteins. Using actin waves that propagate on the cortex of mast cells as a model, we discovered that formins (FMNL1 and mDia3) are recruited before the Arp2/3 complex in actin waves. GTPase Cdc42 interactions drive FMNL1 oscillations, with active Cdc42 and the constitutively active mutant of FMNL1 capable of forming waves on the plasma membrane independently of actin waves. Additionally, the delayed recruitment of Arp2/3 antagonizes FMNL1 and active Cdc42. This antagonism is not due to competition for monomeric actin but rather for their common upstream regulator, active Cdc42, whose levels are negatively regulated by Arp2/3 via SHIP1 recruitment. Collectively, our study highlights the complex feedback loops in the dynamic control of the actin cytoskeletal network.

## In brief

Chua et al. showed that multiple actin nucleators are assembled in cortical actin waves. Their mutual dependency leads to the fine-tuning of actin and the upstream signal transduction networks on the timescale of seconds.

## Graphical abstract

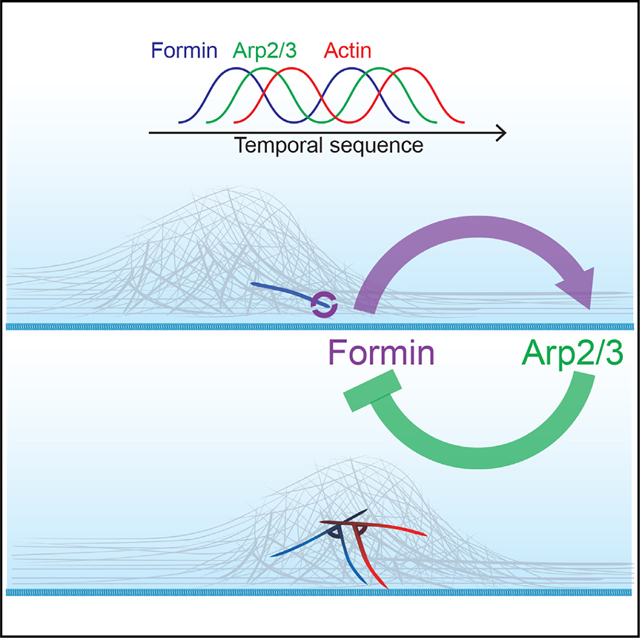

## INTRODUCTION

Cells receive extracellular stimuli to trigger signal transduction pathways and activate the actin cytoskeleton to regulate a plethora of diverse cellular processes, such as cell migration, adhesion, growth, and division. Achieving precise regulation of these processes requires the involvement of a repertoire of proteins capable of orchestrating actin assembly and turnover. Of particular significance are two major classes of actin nucleators: the Arp2/3 complex and formins.^1^ While the simplistic view is that the Arp2/3 complex nucleates the side branching of actin filaments at a 70° angle to allow the growth of new “daughter” filaments, and formins are responsible for the elongation of the barbed ends of linear unbranched actin filaments, increasingly evident is the fact that these nucleators do not work in isolation within cells, but instead frequently interact and generate higher-order cellular architectures. Even though such interplay is well recognized in contexts such as leading-edge assembly during migration, immunological synapses, and adherens junctions,^2,3^ many mechanistic questions remain in regard to their crosstalk.

In recent years, the phenomenon of actin waves has gained widespread attention across diverse cell types, including but not limited to *Dictyostelium*,^4–13^ frog and echinoderm oocytes and embryos,^14–18^ *Caenorhabditis elegans* embryos,^19–21^ *Drosophila* embryo,^22,23^ mouse embryos,^24,25^ mast cells,^26–33^ Jurkat T cells,^34^ neutrophils,^35^ macrophage,^36^ endothelial cells,^37^ and epithelial cells.^38–40^ While classic supramolecular actin assemblies such as lamellipodia and filopodia are dynamic, actin waves involve actin assembly and disassembly in a much more coordinated and transient fashion. A single cycle of assembly and disassembly takes place within a time frame ranging from a few seconds to minutes,^29^ with the fastest oscillatory cycles a mere 8–10 s.^5,8,41^ The underlying rationale for inducing such transient yet recurrent patterns remains a subject of inquiry, with the current literature implicating cortical actin waves in diverse cellular functions, including cell polarity and motility,^42–46^ cell division,^17,18,27,47,48^ cell growth,^49^ cell fate changes,^39^ endocytosis,^30^ phagocytosis,^50–55^ or exocytosis.^36,56^ In addition, intracellular actin waves have been found on mitochondria, regulating membrane dynamics and partition during mitosis.^40,57,58^ Despite certain similarities in the spatial and temporal patterns observed, it is important to recognize the substantial differences characterizing actin waves across diverse systems. These waves are likely heterogeneous in nature and involve distinct higher-order assembly of actin filaments, which remain largely uncharacterized.

In our present work, we employ the actin waves exhibited by mast cells as a model to investigate the nucleation mechanism for actin waves—in particular, how different actin nucleators collaborate within the same system. Our study reveals that both formins, FMNL1 and mDia3, are recruited to actin waves prior to the Arp2/3 complex with a consistent phase shift. The timing of formin recruitment to the membrane is determined by its interaction with GTPase, not with actin. Strikingly, the constitutively active (CA) mutant of FMNL1 can propagate waves even in an F-actin-depleted state. Lastly, we found that acute inhibition of the Arp2/3 complex enhances formin recruitment. This effect does not arise due to the competition for the actin monomer; rather, it is due to the availability of upstream regulators, which is negatively regulated by Arp2/3 via recruitment of the inositol 5-phosphatase SHIP1. Collectively, our analysis reveals the intricate interplay between Arp2/3 and formin-associated actin polymerization in modulating collective actin dynamics through mechanisms involving both cooperation and competition.

## RESULTS

### Arp2/3 complex and formins FMNL1 and mDia3 assemble as coordinated waves

**Figure 1.**
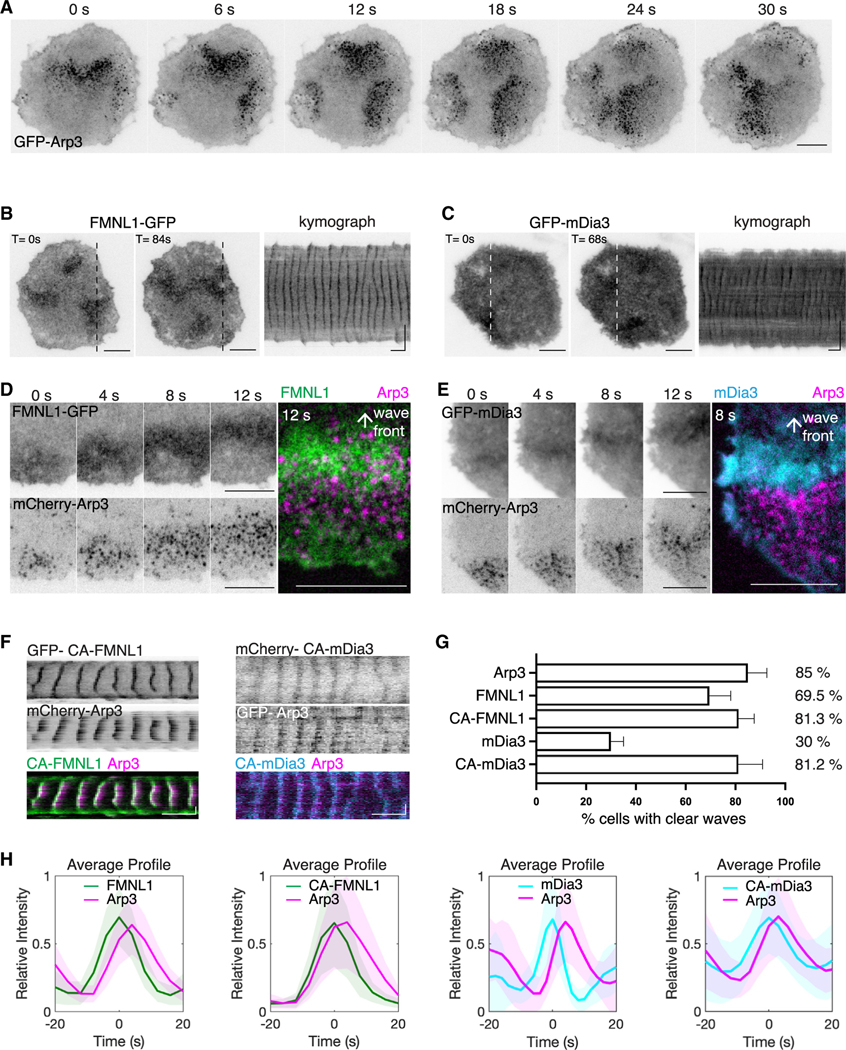
Localization of Arp3, FMNL1, and mDia3 in cortical traveling waves (A) Representative TIRFM time-lapse montage of 6 frames (6-s interval) of a cell expressing GFP-Arp3. (B and C) Representative micrographs and kymographs of a cell expressing FMNL1-GFP (B) or GFP-mDia3 (C). (D and E) Sequential montage of 4 frames (4-s interval) of a region of a representative cell co-expressing FMNL1-GFP and mCherry-Arp3 (D), or GFP-mDia3 and mCherry-Arp3 (E), followed by a 2-color merge micrograph. (F) Representative kymographs of a cell co-expressing (left) GFP-CA-FMNL1 and mCherry-Arp3, or (right) mCherry-CA-mDia3 and GFP-Arp3. (G) Bar plot indicating the percentage of cells exhibiting oscillations when different constructs were expressed (Arp3: 17/19 cells; FMNL1: 17/26 cells; CA-FMNL1: 10/12 cells, 5/19 cells; CA-mDia3: 20/25 cells). Cells were chosen in an unbiased manner from *n* ≥ 2 experiments). Error bars represent the SEM. (H) Representative average profiles of Arp3 aligned with respect to FMNL1-GFP, CA-FMNL1, mDia3, and CA-mDia3. Horizontal scale bars in micrographs: 10 μm. Vertical scale bars in kymographs: 10 μm. Horizontal scale bars in kymographs: 1 min. All grayscale micrographs, kymographs and montages in this paper are shown in the inverted lookup table.

Our previous studies revealed the propagation of cortical oscillatory waves in resting and stimulated rat basophilic leukemia (RBL-2H3) cells, as well as the recruitment of FMNL1, mDia3, and FHDC1 to the ventral actin waves that are Cdc42 dependent.^31^ These three actin nucleators belong to the family of 15 formins, a major class of actin-polymerizing proteins responsible for the elongation of the fast-growing, barbed ends of linear unbranched actin filaments. How they compare to Arp2/3, which generates the branched actin network, is not clear. Here, we examined the dynamics of these actin nucleators, first individually and then in combination. When imaged by total internal reflection fluorescence microscopy (TIRFM), GFP-Arp3 appeared as distinct clusters of puncta that exhibited sequential assembly and disassembly cycles (*n* = 21 cells) (Figure 1A; Video S1). These recurrent cycles of assembly and disassembly were asynchronous at different locations and resembled wavelike movement, but the individual loci did not move in their lifetime. With the maximum intensity projections constructed from the timelapse images, we found out that the probability of Arp2/3 puncta assemblies were relatively uniform throughout the cell, except that they were reduced at the peripheral edges of the cell (Figure S1A). The assembly of Arp2/3 (GFP-Arp3) preceded F-actin (LifeAct-mRuby) in cortical waves (*n* = 28 cells, 10 experiments) (Video S1). To ensure an unbiased analysis of these waves, we generated kymographs at three randomly selected regions of interest (ROIs) within a cell expressing GFP-Arp3 (Figure S1B). At these chosen regions, we also plotted the changes in fluorescence intensities using three different ROI sizes (5 × 5 pixel, 20 × 20 pixel, and 40 × 40 pixel) (Figure S1C). Our analysis showed that the observed wave cycles in the fluorescence intensity plots remained consistent across the three different ROI sizes, as judged by the similar patterns of the rise and decay of each peak. However, peaks of intensity fluctuations acquired from larger ROI size appeared to be smoother than smaller ROI sizes, due to the averaging effect. The number of peaks observed in the plots corresponds to the number of bands in the kymograph re-slices. To maintain consistency, we used an ROI size of 20 × 20 pixel (equivalent to 2.2 × 2.2 μm) for all our data analyses in this study.

Unlike the distinct Arp3 clusters, FMNL1 waves appeared as diffusive but visually apparent clouds (*n* = 33 cells, 13 experiments) (Figure 1B). Compared to FMNL1, mDia3 waves had only a subtle increase in intensity over the background levels, resulting in a less distinct contrast (*n* = 28 cells, 10 experiments) (Figure 1C). To confirm that mDia3 waves were a result of their recruitment to the plasma membrane, not simply an artifact caused by membrane fluctuations that could lead to changes in TIRF signals, we co-expressed GFP-mDia3 with a plasma membrane marker (PM-mScarlet). In cells that exhibited waves of mDia3, we did not observe intensity oscillations of plasma membrane marker at the same amplitude and regularity, indicating that the observed waves of formins could not be caused by membrane lifting from the TIRF plane (*n* = 12/12 cells in 2 experiments) (Figure S2).

To gain an understanding of the nucleation process driving the actin wave formation, we visualized the dynamics between these two classes of nucleators. To this end, we co-expressed the fluorescently labeled marker for either FMNL1 or mDia3 with mCherry-Arp3. We found that the waves of FMNL1 and Arp3 always appear in the same cells (Figure 1D; Video S2). In these cells, waves of Arp3 trailed behind the corresponding waves of FMNL1 (*n* = 29 cells, 11 experiments) (Figure 1D). In contrast, mDia3 only appeared as waves with Arp3 in a subset of the cells (Figure 1E). Given the auto-regulatory properties of diaphanous-related formins, we also tested the effect of releasing formins from their auto-inhibited states. The L1062D mutation at the diaphanous auto-regulatory domain (DAD) motif of FMNL1 prevents its interaction with the diaphanous inhibitory domain (DID) motif, thus rendering it CA.^59^ For mDia3, we deleted the DAD domain to generate a CA mutant of mDia3. We co-expressed CA mutants of mDia3 (mCherry-CA-mDia3) and Arp3 (GFP-Arp3) in cells and observed an increased appearance of waves of CA-mDia3 (*n* = 11 cells, 2 experiments) (Figure 1F). CA-FMNL1 also displayed a modest increase in the percentage of cells showing regular oscillation (*n* = 9 cells, 3 experiments). We quantified fluorescence intensities using fast Fourier transform (FFT) and used the presence of frequency peak (in the range of 5–100 s) as a criteria. FMNL1, CA-FMNL1, mDia3, and CA-mDia3 displayed oscillatory wave patterns in 69.5%, 81.3%, 30%, and 81.2% of the cells counted (randomly chosen cells: Arp3, 17/19 cells; FMNL1, 17/26 cells; CA-FMNL1, 10/12 cells, 5/19 cells; CA-mDia3, 20/25 cells), respectively (Figure 1G). Average intensity profiles acquired from the co-expression of Arp3 with either formin construct showed that CA mutants shared a similar temporal phase as their wild-type proteins and that FMNL1, CA-FMNL1, mDia3, and CA-mDia3 were recruited ahead of Arp3 in cortical wave propagation (FMNL1 precedes Arp3 by 2.3 ± 0.7 s [*n* = 30 cells, 13 experiments]; CA-FMNL1 precedes Arp3 by 2.1 ± 0.3 s [*n* = 10 cells, 4 experiments]; mDia3 precedes Arp3 by 4.2 ± 1.5 s [*n* = 24 cells, 7 experiments]; and CA-mDia3 precedes Arp3 by 1.9 ± 0.9 s [*n* = 14 cells, 2 experiments]) (Figure 1H). Our results collectively illustrate the variations in the distributions of actin nucleators. Nonetheless, the recruitment of formins (FMNL1 and mDia3) always preceded Arp2/3 complex.

### FMNL1 recruitment to waves requires the presence of a functional GTPase binding domain (GBD) and the alleviation of auto-inhibition interactions

**Figure 2.**
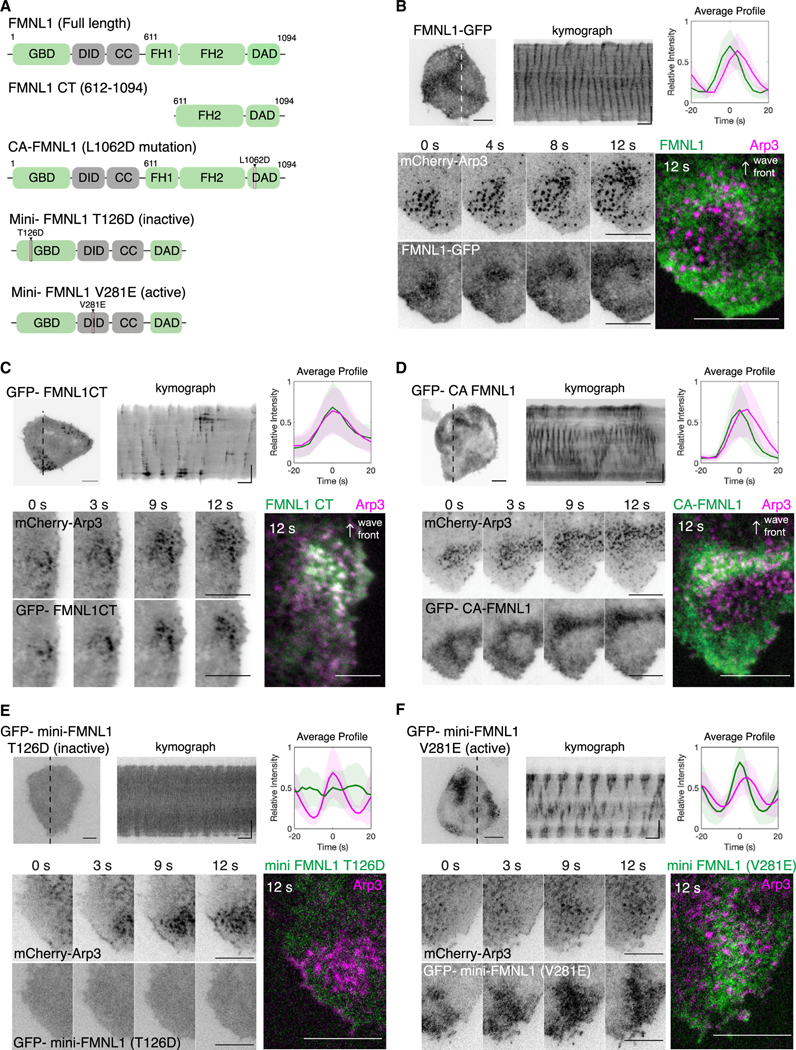
Recruitment of FMNL1 and FMNL1 mutants relative to Arp3 in cortical traveling waves (A) Domain schematics of various FMNL1 constructs used in this study. (B–F) Representative micrographs, kymographs, intensity profiles, and sequential montage of 4 frames (3- to 4-s interval) of a region of a cell co-expressing mCherry-Arp3 and (B) FMNL1-GFP, (C) GFP-FMNL1CT, (D) CA-FMNL1, (E) GFP-mini-FMNL1-T126D (inactive mutant), or (F) GFP-mini-FMNL1-V281E (active mutant). Horizontal scale bars in micrographs: 10 μm. Horizontal scale bars in kymographs: 1 min. Vertical scale bars in kymographs: 10 μm. Representative average profiles of mCherry-Arp3 aligned with respect to FMNL1 constructs.

To gain further insights into how FMNL1 was recruited to the membrane, we employed a range of truncated and point mutants of FMNL1 (Figure 2A)^59^ and compared them with Arp3. FMNL1 shares the conserved domain architecture with the diaphanous family of formins, defined by their N terminus GBD, followed by the DID, coiled-coil, dimerization domain, formin homology-1 (FH1) and −2 (FH2) domains, and finally, the DAD at the C terminus (Figure 2A). While the waves of GFP-FMNL1 were recruited before Arp3 and did not co-cluster with Arp3 (Figure 2B; Video S2), the C termini mutant of FMNL1 with the FH2 and DAD motifs (GFP-FMNL1CT) appeared as punctate structures (Figure 2C; Video S3). These punctate structures of FMNL1CT co-localized spatially and temporally with the Arp3 puncta (*n* = 15/15 cells, 2 experiments). Waves exhibited by the FMNL1CT were irregular and propagated only in localized regions of the cell, in contrast with cells co-expressing mCherry-Arp3 with FMNL1 (Figures 2B and 2C; Video S2). Our cross-correlation analysis revealed a minimal phase difference between FMNL1CT and Arp3 (0.3 ± 0.7 s, *n* = 15/15 cells, 2 experiments) (Figure 2C). Similar to the waves exhibited by FMNL1, GFP-CA-FMNL1 waves were diffuse, despite exhibiting heterogeneity in the regularity of their wave-like movements (Figures 1B, 1F, and 2D; Video S2 and S4). These results suggest that the C termini of FMNL1 and how they interact with actin unlikely explain the early recruitment of FMNL1.

To test whether the actin-interacting FH domains of FMNL1 were necessary, we next conducted experiments using two “mini” mutants of FMNL1 whose FH1-FH2 domains were substituted by a (Gly-Gly-Ser)_2_ linker.^59^ The first mutant, mini-FMNL1-T126D, is deficient in its binding to its endogenous activating GTPase, and the second mutant, mini-FMNL1-V281E, is deficient in DAD binding and mimicks an active conformation.^59^ GFP-mini-FMNL1-T126D did not exhibit any observable wave-like behavior, despite the clear wave of Arp3 co-expressed in the same cell (*n* = 16 cells, 4 experiments) (Figure 2E). In contrast, GFP-mini-FMNL1-V281E was observed to assemble into clear waves and precede Arp3 waves by 2.5 ± 0.65 s (*n* = 14 cells, 2 experiments) (Figure 2F), similar to the temporal dynamics exhibited by FMNL1 or CA-FMNL1. Taken together, these results illustrate the order in which the membrane and actin interaction sites orchestrate the temporal recruitment and action of FMNL1. The GTPase-interaction domain is not only essential for the recruitment of FMNL1 to the membrane but it is also responsible for its timing. In the absence of the GTPase-binding domain, the expression of the FH2 and DAD motifs of FMNL1 alone allows its recruitment to waves, but it results in altered phase, localization, and wave geometry.

### Membrane waves of active Cdc42 and CA FMNL1 continued in the absence of F-actin waves

**Figure 3.**
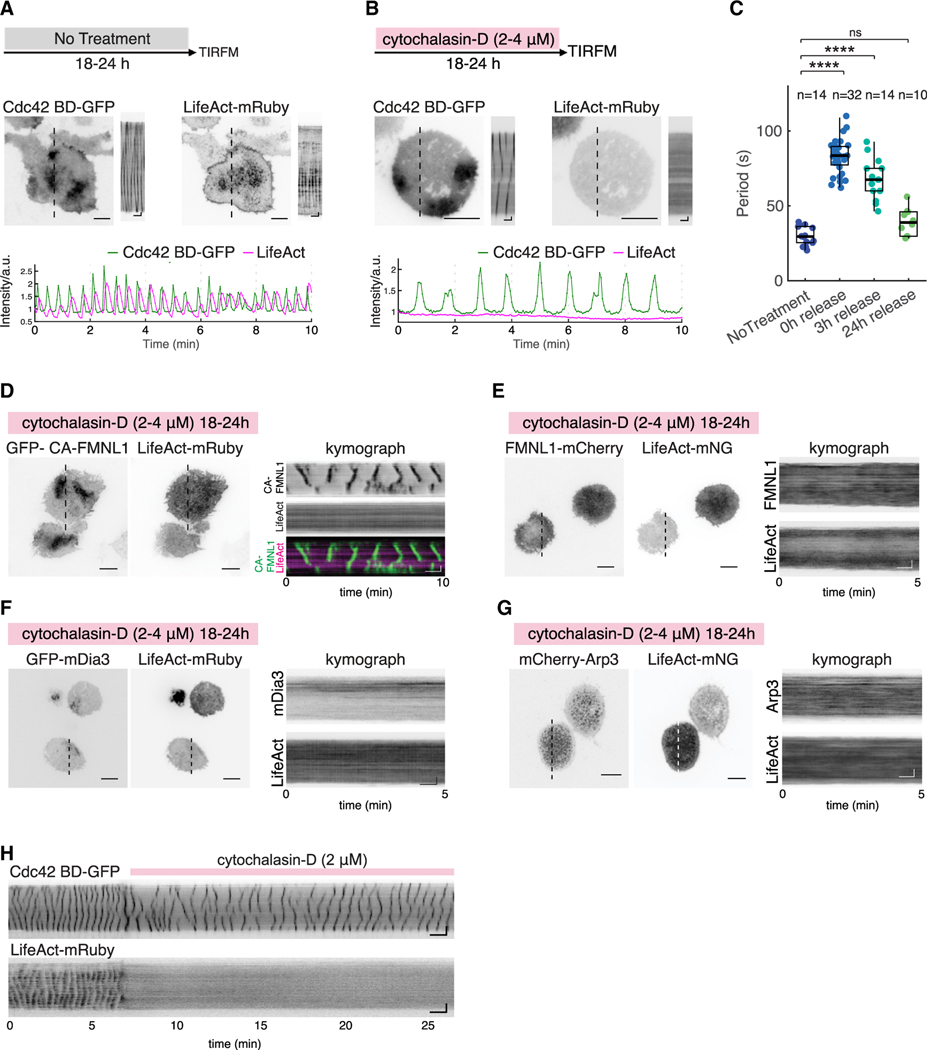
Cortical waves of active Cdc42, formins, and Arp3 in response to cytochalasin-D treatment (A and B) Representative micrographs, kymographs, and intensity plot of a cell stably expressing Cdc42 BD-GFP co-transfected with LifeAct-mRuby under (A) untreated condition or (B) F-actin depleted condition with cytochalasin-D. (C) Box and whisker plots comparing the periodicities of active Cdc42 oscillations in cytochalasin-D-pretreated cells of different release periods are shown. *n* indicates number of cells imaged. Box: 1st/3rd quartiles; whisker: 2nd and 98th percentile. Statistical differences: ****p < 0.0001; ns, not significant, 1-way ANOVA, Tukey’s multiple comparison tests. (D–G) Representative micrographs and kymographs of cells treated with cytochalasin-D (2–4 μM) over 18–24 h co-expressing LifeAct-mRuby or LifeAct-mNeonGreen with (D) GFP-CA-FMNL1, (E) FMNL1-mCherry, (F) GFP-mDia3, or (G) mCherry-Arp3. (H) Kymograph of a cell before and after treatment with cytochalasin-D (2 μM). Horizontal scale bars in micrographs: 10 μm. Vertical scale bars in kymographs: 10 μm. Horizontal scale bars in kymographs: 1 min. Cytochalasin-D inhibitor experiments were performed in normal growth medium.

If GTPase-binding determines the phase of FMNL1 recruitment prior to Arp2/3, we next examined whether this interaction is sufficient and FMNL1 could oscillate independently of actin oscillations. We imaged cells expressing a Cdc42 biosensor (Cdc42 BD-GFP), a reporter for the endogenous GTPase activity of active Cdc42, and LifeAct-mRuby, which serves as as a reporter for actin. Cells co-expressing both markers exhibited sustained waves over time (Figure 3A). We sought to identify experimental conditions that would reduce or eliminate actin oscillation. To achieve this, we used cytochalasin-D, a pharmacological inhibitor that disrupts the actin cytoskeleton by blocking the barbed ends of actin filaments.^60^ Through systematic experimentation of varying drug doses and incubation time, we found that treatment with cytochalasin-D (2–4 μM) over a period of 18–24 h could uncouple active Cdc42 oscillations from actin oscillations (Figures 3B and S3A). Cells exhibited clear waves of active Cdc42 in the absence of detectable LifeAct waves (*n* = 32 cells, 3 experiments) (Figure 3B; Video S5). The observed waves of active Cdc42 had a longer oscillatory cycle time compared to waves in untreated conditions (Figures 3A–3C; Video S6). Elimination of actin waves was reversible. If the cytochalasin-D-treated cells were allowed to recover in fresh growth media, actin waves would resume over time (Figure S3B). Pre-treated cells released into fresh growth media for 3 and 24 h exhibited periodicities of 67.9 ± 13.4 and 39.3 ± 9 s, respectively (Figures 3C, S3B, and S3C). We then proceeded to examine other markers by treating cells with cytochalasin-D overnight and imaged them without release. Under this condition, GFP-CA-FMNL1 exhibited clear waves in the absence of LifeAct-mRuby waves (*n* = 14/14 cells, 3 experiments) (Figures 3D; Video S7). However, waves of Arp3, FMNL1, or mDia3 were abolished when LifeAct waves were undetectable, and all could resume in the recovery phase when LifeAct waves came back (*n* = 20 cells co-expressing mCherry-Arp3 and LifeAct-mNG in 2 experiments; *n* = 7 cells co-expressing FMNL1-GFP and LifeAct-mRuby in 2 experiments; *n* = 11 cells co-expressing GFP-mDia3 and LifeAct-mRuby in 2 experiments) (Figures 3E–3G). Similar effects could be observed when cytochalasin-D (2 μM) was administered acutely (Figures 3H and S4). However, while LifeAct oscillations were observed to be abolished immediately in all cells, the effects on active Cdc42 oscillations were more heterogeneous with this acute cytochalasin-D treatment. Cells could transiently stop waves, but recover a few minutes later (Figure S4A) or abolish both LifeAct and active Cdc42 waves together (Figure S4B). Collectively, our results showed that the propagation of Cdc42 and CA-FMNL1 waves can be independent of actin waves. While membrane-dependent interactions are sufficient to drive CA-FMNL1 recruitment and oscillations, this interaction is not strong enough for FMNL1, which is likely in its auto-inhibited state.

### Synergy of FMNL1 and Arp2/3 complex assembly in waves

**Figure 4.**
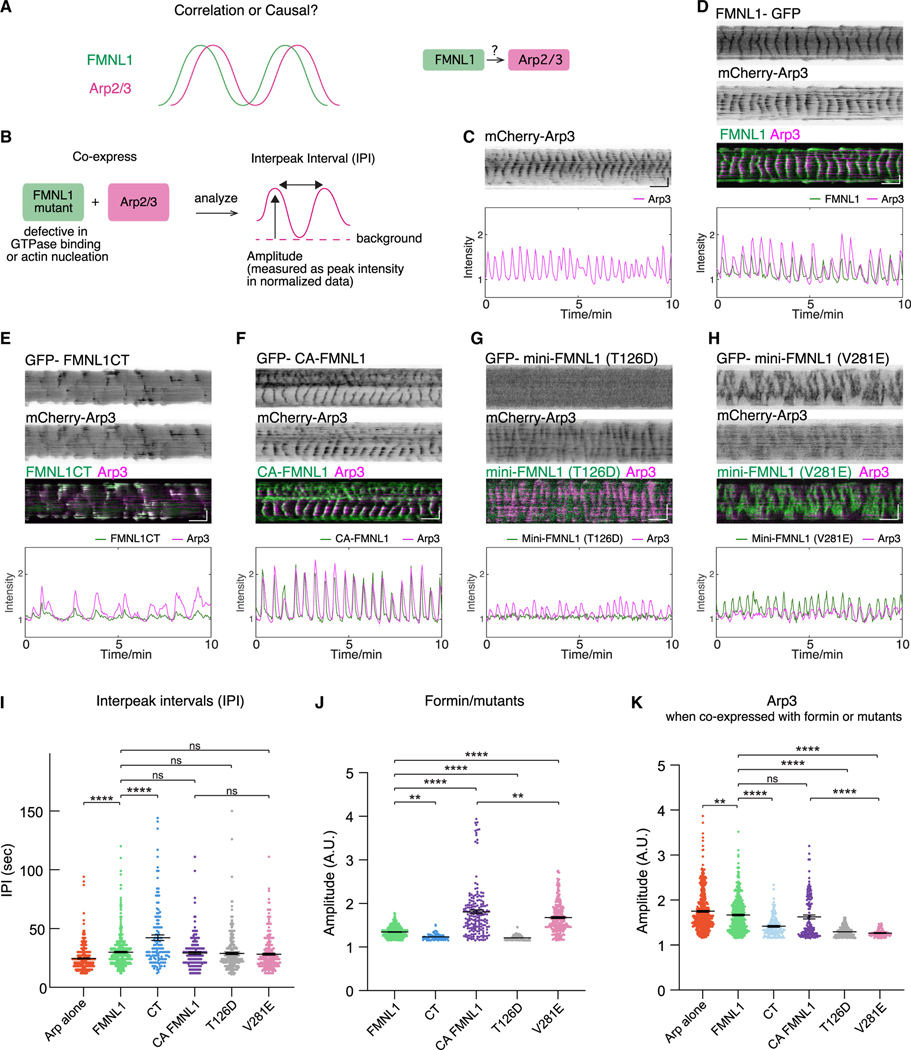
Effects of expressing FMNL1 and FMNL1 mutants on Arp3 waves (A) Illustration of the relative phases of FMNL1 and Arp2/3 and whether an upstream role of FMNL1 relative to Arp2/3 exists. (B) Schematic illustrating the analysis of average peak amplitudes and IPI exhibited by the co-expression of FMNL1 or different FMNL1 mutants with mCherry-Arp3. (C) Representative kymograph and intensity profile of a cell expressing mCherry-Arp3. (D–H) Representative kymograph and intensity profile of a cell co-expressing mCherry-Arp3 and (D) FMNL1-GFP, (E) GFP-FMNL1CT, (F) GFP-CA-FMNL1, (G) GFP-mini-FMNL1-T126D (inactive mutant), or (H) GFP-mini-FMNL1-V281E (active mutant). (I) Quantifications for the average IPI of cells expressing mCherry-Arp3 alone or co-expressing mCherry-Arp3 with different formins. (J) Quantifications for the average peak amplitudes of formin constructs. (K) Quantifications for the average peak amplitudes of Arp3 when cell expresses mCherry-Arp3 or co-expresses mCherry-Arp3 with formin constructs. Sample sets for (J) and (K): single expression of mCherry-Arp3: 15 cells. Co-expression of mCherry-Arp3 with the following: FMNL1-GFP: 22 cells; GFP-FMNL1CT: 16 cells; GFP-CA-FMNL1: 9 cells; GFP-mini-FMNL1-T126D (inactive mutant): 16 cells; GFP-mini-FMNL1-V281E (active mutant): 14 cells. Statistical significance: ****p < 0.0001; ns, not significant; **p < 0.012–0.0055, 1-way ANOVA, Dunnett’s multiple comparison test. Error bars represent mean ± SEM.

Observing the synchronized patterns of Arp2/3 and FMNL1 waves, with FMNL1 waves preceding Arp3 temporally, we were prompted to investigate whether FMNL1 operates upstream of Arp2/3 in actin wave dynamics, or whether their correlation merely suggests parallel pathways with no direct causality between these two nucleators (Figure 4A). To this end, we analyzed the impact of expressing FMNL1 mutants on the oscillation pattern of mCherry-Arp3 (Figure 4B). As a control, we performed single expression experiments of mCherry-Arp3 (*n* = 15 cells, 3 experiments) (Figure 4C). Given that several mutants exhibited irregular fluctuations (Figures 4D–4H), we proceeded to analyze individual peaks (defined by local maximal signals) and the interpeak intervals (IPIs), as well as the distribution of peak intensity (Figure 4B). The average IPI of Arp3 when expressed alone was faster (24.6 ± 9.9 s), while the rest were comparable, with the exception of FMNL1CT (FMNL1: 30 ± 13.3 s; FMNL1CT: 42.2 ± 26.7 s; CA-FMNL1: 29.2 ± 14 s; mini-FMNL1-T126D [inactive]: 28.9 ± 15.2 s; mini-FMNL1-V281E [active]: 28.2 ± 14.6 s) (Figures 4I and S5). Our quantification showed that both active mutants (CA-FMNL1 and mini-FMNL1-V281E) had higher peak intensity as compared to FMNL1 (FMNL1: 1.3 ± 0.1; CA-FMNL1: 1.8 ± 0.6; mini-FMNL1-V281E [active]: 1.7 ± 0.4, *p* < 0.0001) (Figures 4J and S5). However, the FMNL1 mutants without their membrane binding, such as the FMNL1CT and mini-FMNL1-T126D (inactive) mutants, exhibited lower peak amplitudes, as compared to FMNL1 (FMNL1CT: 1.2 ± 0.1, *p* = 0.0055; mini-FMNL1-T126D [inactive]: 1.2 ± 0.1, *p* < 0.0001) (Figures 4J and S5). For Arp2/3 oscillations, there are two groups. First, the peak amplitudes of Arp3 were comparable when expressed alone, but they exhibited a modest drop when co-expressed with either FMNL1-GFP or GFP-CA-FMNL1 (mCherry-Arp3 alone: 1.8 ± 0.1; with FMNL1: 1.7 ± 0.4, *p* = 0.0012; with CA mutant of FMNL1: 1.6 ± 0.5, *p* = 0.0024) (Figures 4K and S5). Second, the average peak amplitudes of Arp3 were reduced significantly when co-expressed with the FMNL1 mutants deficient in membrane binding or actin nucleation, such as the FMNL1CT, mini-FMNL1-T126D (inactive), and mini-FMNL1-V281E (active), when compared to peak amplitudes when co-expressed with FMNL1 (FMNL1: 1.7 ± 0.4; FMNL1CT: 1.4 ± 0.2; mini-FMNL1-T126D [inactive]: 1.3 ± 0.1; mini-FMNL1-V281E [active]: 1.4 ± 0.2, *p* < 0.0001) (Figures 4K and S5). Importantly, while the two active mutants CA-FMNL1 and mini-FMNL1-V281E (deficient of its actin nucleation capacities) exhibited similar peak amplitudes with no statistical differences (Figure 4J), the amplitudes of Arp2/3 were greatly reduced in the presence of mini-FMNL1-V281E (Figure 4K), suggesting that the actin nucleation capability of FMNL1 is likely important for the amount of Arp2/3 recruited. The inactive mini-FMNL1-T126D mutant did not participate in waves, but it subdued the Arp3 amplitude, suggesting a dominant-negative effect on Arp2/3.

### Arp2/3 complex assembly antagonizes formins

**Figure 5.**
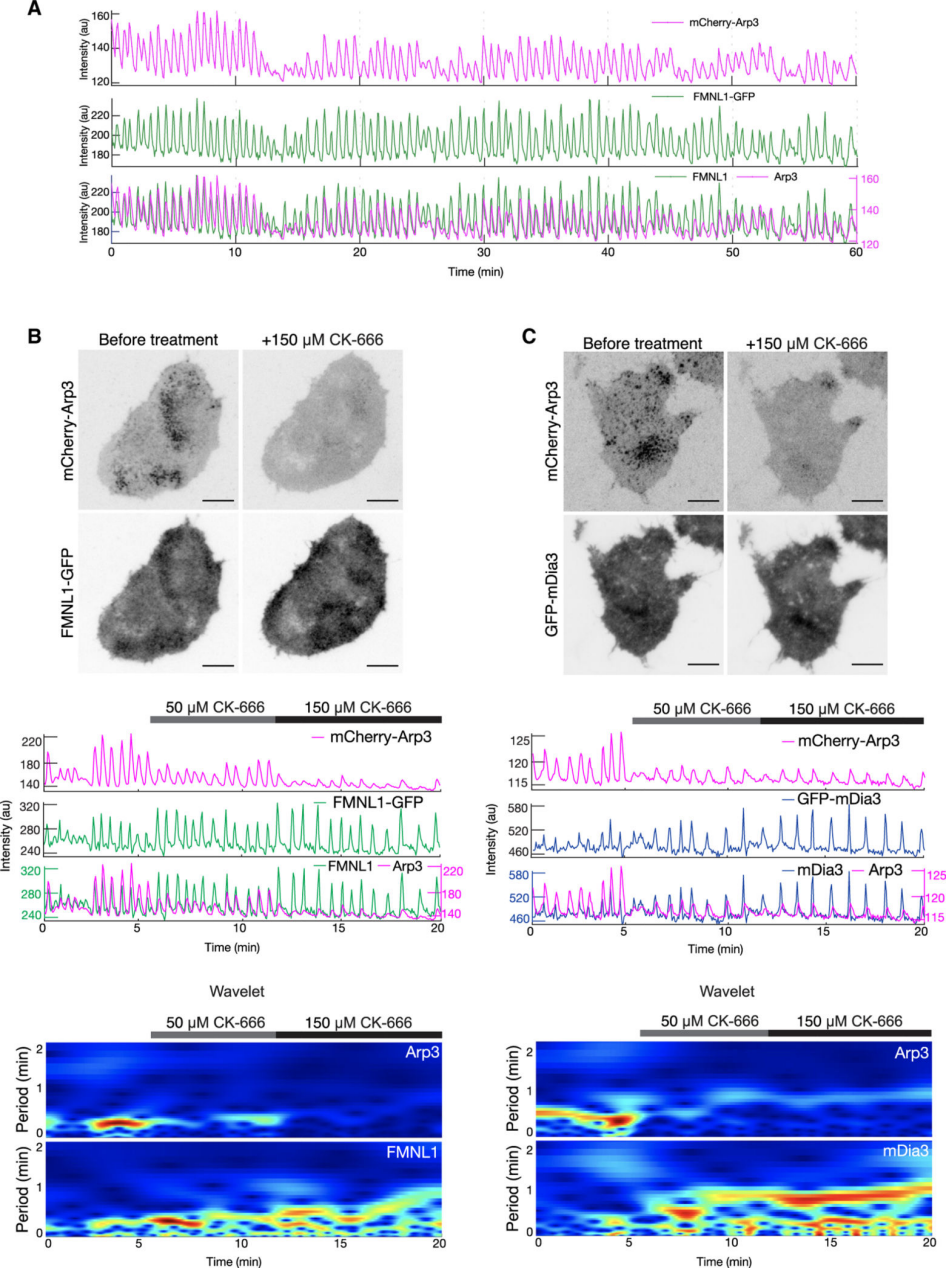
Effects of Arp2/3 inhibition on formin waves (A) Representative intensity profile of a cell co-expressing mCherry-Arp3 and FMNL1-GFP over an acquisition period of 1 h. (B) (B and C) Representative micrographs (top), intensity plots (center), and wavelet analysis (bottom) of a cell co-expressing mCherry-Arp3 and FMNL1-GFP (B), or mCherry-Arp3 and GFP-mDia3 (C) before and after treatment with CK-666. Scale bars: 10 μm. CK-666 inhibitor experiments were performed in Tyrode’s imaging buffer.

Our results thus far reveal the importance of FMNL1 assembly prior to the recruitment of the Arp2/3 complex. Consistent with this, when we monitored FMNL1-GFP and mCherry-Arp3 over extended durations (30–60 min), the amplitudes of FMNL1 and Arp3 oscillations showed strong coordination (*n* = 14 cells, 8 experiments) (Figure 5A). We next investigated the role of Arp3 in cortical waves and, more specifically, the effect of inhibiting Arp2/3 on FMNL1 waves. When treated with CK-666, a pharmacological inhibitor of the Arp2/3 complex,^61^ most of the mCherry-Arp3 puncta disappeared along with the Arp3 oscillations, but not FMNL1 (Figure 5B). Surprisingly, the intensities of FMNL1-GFP waves were enhanced upon treatment with CK-666 at 50 and 150 μM (50 μM CK-666: *n* = 11 cells; 150 μM CK-666: *n* = 3 cells) (Figure 5B; Video S8). A similar antagonistic relationship was found between GFP-mDia3 and mCherry-Arp3 (50 μM CK-666: *n* = 7 cells; 150 μM CK-666: *n* = 7 cells) (Figure 5C; Video S9). The oscillatory amplitudes of mDia3 increased in response to CK-666 treatment at 50 and 150 μM (Figure 5C). Moreover, we also observed an increase in the oscillatory periodicities, as indicated in the wavelet analysis (50 CK-666: *n* = 18 cells; 150 μM CK-666: *n* = 10 cells) (Figure 5C). These results suggest that the delayed recruitment of Arp2/3 may inhibit the recruitment or accumulation of formins.

### Arp2/3 as clusters of puncta with FBP17, N-WASP, and SHIP1

**Figure 6.**
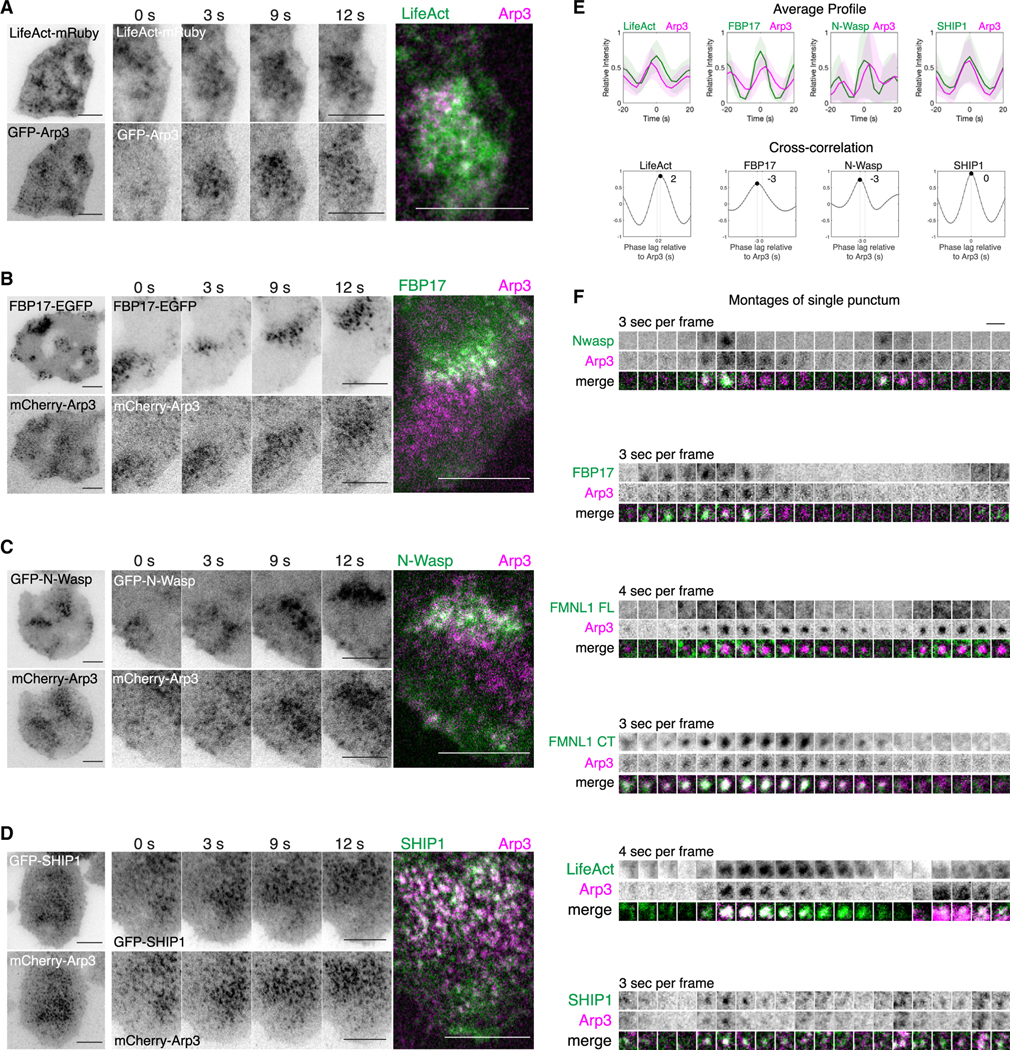
Cluster formation of Arp2/3 puncta and other actin-associated proteins in traveling waves (A–D) Representative micrographs, sequential montage of 4 frames (3-s interval) of a region of a cell co-expressing either GFP-Arp3 or mCherry-Arp3 with (A) LifeAct-mRuby, (B) FBP17-EGFP, (C) GFP-N-WASP, or (D) GFP-SHIP1. (E) Representative average profiles of Arp3 aligned with respect to LifeAct, FBP17, N-WASP, and SHIP1, and the corresponding cross-correlation analyses. (F) Sequential montages of a single punctum of 20 frames (3- to 4-s interval) of a cell co-expressing either GFP-Arp3 or mCherry-Arp3 with LifeAct-mRuby, FBP17-EGFP, GFP-N-WASP, and GFP-SHIP1. Scale bars in (A)–(D): 10 μm. Scale bar in (F): 2 μm.

To understand how Arp2/3 may play a negative role in formin assembly, we examined additional proteins associated with Arp2/3-clusters. We first co-expressed GFP-Arp3 and LifeAct-mRuby to observe the localizations and dynamics of both proteins. Similar to the clusters of Arp3 puncta, LifeAct-mRuby appeared as puncta of F-actin, which co-localize and form coordinated waves along the membrane with the Arp3 clusters, indicating that the Arp2/3 clusters were associated with the increased density of F-actin (Figure 6A). FBP17 and N-WASP are two known activators of Arp2/3-mediated actin polymerization.^62–68^ FBP17 is an F-BAR membrane-binding protein, which activates N-WASP, the direct activator of the Arp2/3 complex for the recruitment of F-actin. When co-expressed with mCherry-Arp3, FBP17-EGFP and GFP-N-WASP appeared to be clustered and preceded mCherry-Arp3 in waves (Figures 6B and 6C). SHIP1 is an inositol 5-phosphatase shown to operate on the negatively charged phosphoinositide (PI) PI(3,4,5)P_3_, which recruits actin-binding proteins to the membrane. In our previous study, we observed the localization of SHIP1 as clusters of puncta that trailed behind the waves of FBP17, defining the refractory phase for PI(3,4,5)P_3_-FBP17 oscillations.^33^ The clusters of punctas for GFP-SHIP1 and mCherry-Arp3 co-localized spatially and temporally (Figure 6D). To characterize the temporal relationship between these proteins, we quantified the cross-correlation of the acquired intensity profiles of each pair of proteins over time. Our results revealed that Arp3 precedes LifeAct by 2.32 ± 1.12 s (*n* = 28 cells, 9 experiments), whereas FBP17 and N-WASP precede Arp3 by 2.63 ± 0.52 s (*n* = 8 cells, 3 experiments) and 2.78 ± 0.94 s (*n* = 18 cells, 7 experiments), respectively (Figure 6E). Arp3 precedes SHIP1 by 0.2 ± 0.41 s (*n* = 15 cells, 3 experiments) (Figure 6E). Despite the lack of perfect colocalization due to time delay, these markers were sequentially recruited to the same cluster, with the exception of FMNL1, which was recruited in the same neighborhood but was not clustered (Figure 6G).

### FMNL1 and Arp2/3 competition through a limited pool of active Cdc42

**Figure 7.**
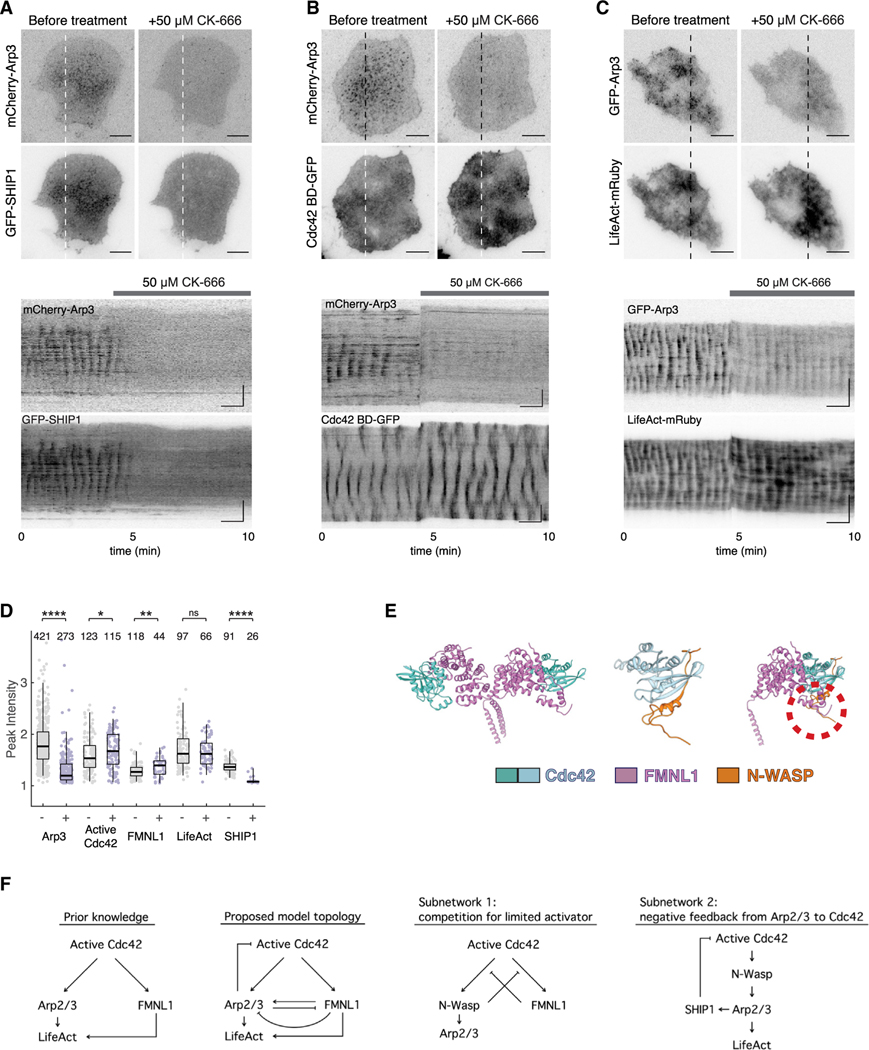
Cortical waves of F-actin, active Cdc42, and SHIP1 in response to Arp2/3 inhibition (A–C) Representative micrographs (top) and kymographs (bottom) of a cell co-expressing either GFP-Arp3 or mCherry-Arp3 with (A) GFP-SHIP1, (B) Cdc42 BD-GFP, or (C) LifeAct-mRuby before and after treatment with 50 μM CK-666. (D) Box and whisker plots for the average peak oscillatory amplitudes exhibited by either Arp3 (57 cells) with active Cdc42 (19 cells), FMNL1 (17 cells), LifeAct (14 cells), or SHIP1 (7 cells) before (−) and after (+) treatment with 50 μM CK-666 are shown with the number of data points shown above each plot. *n* indicates the number of peaks. Box: 1st/3rd quartiles; whisker: 2nd and the 98th percentile. Statistical significance: ****p < 0.0001; **p = 0.0011; *p = 0.0246; ns, not significant, t test. Error bars represent mean ± SEM. CK-666 inhibitor experiments were performed in Tyrode’s imaging buffer. (E) Structure of the dimeric Cdc42/FMNL1 complex (PDB: 4ydh), Cdc42/N-WASP complex (PDB: 1cee), and Cdc42/N-WASP complex aligned with Cdc42/FMNL1 complex on the basis of Cdc2 using chimera. Models of Cdc42 are shown in light blue and turquoise, model of the GTPase binding domain of N-WASP is shown in orange, and the model of the N-terminal domain of FMNL1 is shown in purple. Red dashed circle shows the region where FMNL1 and N-WASP clash. (F) Prior and updated model on the crosstalk and feedback mechanisms in the Cdc42-mediated actin wave network.

Cdc42 is a shared upstream regulator for FMNL1 and Arp2/3. Cdc42 GTPase regulates Arp2/3-mediated actin polymerization through the activation of N-WASP/WASP, which subsequently activates the Arp2/3 complex. Furthermore, previous studies have shown that Cdc42 can form complexes with both WASP and FMNL1.^69^ Because SHIP1 is a lipid phosphatase hydrolyzing PI(3,4,5)P_3,_ which regulates the recruitment of Cdc42, Arp2/3 may recruit SHIP1 and regulate Cdc42 through a negative feedback mechanism. Indeed, we observed the abolishment of both waves of Arp3 and SHIP1 when treated with CK-666 at 50 μM (*n* = 7 cells, 2 experiments) (Figure 7A). The period of cortical oscillations depends on the levels of PI(3,4,5)P_3_.^33^ Therefore, the reduction in SHIP1 clusters is consistent with increased oscillatory period when Arp2/3 levels are inhibited (Figure 6D). Next, we examined the level of active Cdc42 under the same conditions. Consistent with the loss of SHIP1, treating cells co-expressing Cdc42 BD-GFP and mCherry-Arp3 with 50 μM CK-666 lead to increased active Cdc42 activity (*n* = 12 cells, 4 experiments) (Figure 7B; Video S10). Considering the increase in the membrane recruitment of formins in response to reduced Arp2/3-mediated actin polymerization, we wondered if the levels of F-actin were perturbed under the same treatment conditions, with actin waves as a readout. When Arp3 puncta and waves were perturbed after treatment with CK-666 at 50 μM, we saw an increase in the intensities of F-actin waves (LifeAct-mRuby) (*n* = 10 cells, 4 experiments) (Figure 7C). At a higher dosage of CK-666, at 200 μM, we observed the inhibition of both LifeAct and Arp3 waves (*n* = 2 cells, 2 experiments) (Figure S6A). Further increases in the concentration of CK-666 to 250 μM completely abolished both LifeAct and Arp3 waves (*n* = 4 cells, 3 experiments) (Figure S6B). Surprisingly, we noticed that the enhanced waves of active Cdc42 sustained throughout the acquisition period, even after treatment with CK-666 of either 250 μM (*n* = 8 cells, 4 experiments) or 500 μM (*n* = 4 cells, 2 experiments) (Figure S6C). Similarly, the waves of FMNL1 persisted even when treated with CK-666 at the highest dosage of 250 μM (*n* = 8 cells, 3 experiments) but reduced at 500 μM (*n* = 6 cells, 2 experiments) (Figure S6D). The increase in the oscillatory amplitudes exhibited by active Cdc42 and FMNL1 and the decrease in SHIP1 level in response to Arp2/3 inhibition were statistically significant (active Cdc42: 1.6 ± 0.3–1.7 ± 0.3; FMNL1: 1.3 ± 0.1–1.4 ± 0.2; SHIP1: 1.4 ± 0.1–1.1 ± 0.1) (Figures 7D and S7). Furthermore, an analysis of the available structures of the Cdc42/WASP and Cdc42/FMNL1 complexes revealed significant steric clashes between these two complexes (Figure 7E). Taken together, these results reveal an increase in formin recruitment and actin polymerization in response to intermediate doses of Arp2/3 inhibition by CK-666. Arp2/3 complex, which was recruited after formin, appear to limit formin-mediated actin polymerization in actin waves. Our quantitative data are consistent with a model where the antagonism between Arp2/3 complex and formins is regulated more upstream, likely involving their competition for active Cdc42, which in turn is limited by Arp2/3-dependent recruitment of SHIP1 (Figure 7F).

## DISCUSSION

Directly visualizing both groups of nucleators under the same physiological conditions has posed challenges. Detecting formin activity in living cells often requires the use of CA mutants, which can disrupt the physiological cytoskeletal network and actin dynamics. Genetic perturbations tend to be too slow and are limited in dissecting systems with multiple levels of positive and negative feedback loops. Actin waves offer an ideal experimental model for studying collective dynamics. Here, we successfully visualized the dynamics of these crucial actin regulators under physiological conditions with high spatial and temporal resolution. This enables us to quantitatively characterize the complex, multilayered interplay, encompassing both coordination and competition.

### Role of Arp2/3 in actin waves

We were surprised to discover that Arp2/3 follows formin waves, and inhibiting the majority of Arp2/3 minimally affects the amplitudes of actin oscillations in Cdc42-dependent waves. Our data do not dispute the essential role of Arp2/3 in nucleating actin waves. Arp2/3 recruitment to Cdc42 was observed in every cell with Cdc42 and actin waves. In contrast, FMNL1 waves were present in the majority of cells with Cdc42 waves, while mDia3 waves appeared in only a subset of cells with Cdc42 waves. Also, at high concentrations of CK-666 where Arp2/3 oscillations were not detectable, actin waves were also inhibited, even though waves of Cdc42 and FMNL1 persisted, suggesting the necessity of Arp2/3 in polymerizing actin waves or activating formin activity.

Nonetheless, these findings are unexpected, given that Arp2/3 is the most well-established downstream effector for Cdc42 and N-WASP. Activating actin polymerization by Cdc42 through N-WASP and Arp2/3 is a classic pathway that has been investigated extensively.^68,70^ These studies demonstrated both the necessity and the sufficiency of Arp2/3 in Cdc42-regulated actin polymerization. Necessity was proved by depleting Arp2/3^71^ or N-WASP in the extract-based reconstitution assays for Cdc42-dependent actin polymerization,^66,72^ and sufficiency was shown by *in vitro* actin polymerization assays using purified Arp2/3 with N-WASP^66^ or SCAR.^73^ These interactions of Cdc42 and WASP are also supported by structural evidence.^69,74^ However, Arp2/3 was not identified through an unbiased screen for the most potent actin nucleator. Data derived from Arp2/3 knockout cells have suggested that the Arp2/3 complex is not as ubiquitously essential for actin dynamics as originally thought.^75–77^ Our data are consistent with the emerging view that Arp2/3 nucleated actin branches could be more specialized. Arp2/3 network was proposed to form in response to mechanical cues,^78,79^ and the Arp2/3 generated actin network was also found to display different mechanical properties.^80–83^ Since the basal membrane is connected to the substrate, the recruitment of Arp2/3 may be related to the adhesion state of the cortex. Within the single cell, we found that Arp2/3 was preferentially localized in the cell center relative to the cell edge, which could be related to the adhesion state of the cell center. In addition, actin waves after Arp2/3 inhibition persisted, but they have a longer cycle time. These suggest that the function of Arp2/3 could be to promote faster turnover of actin filaments, thereby modulating the instability of these actin networks in a location- and dynamics-dependent manner. This regulatory role of the kinetics could be related to the formation of Arp2/3 puncta, which subsequently clustered other actin-binding proteins, including lipid phosphatase SHIP1.

### Coordination between Arp2/3 and formins

In the resting cortex of interphase HeLa cells, it was estimated that about 10% of cortical actin was nucleated by formins.^81^ The much higher relative contributions of formin-mediated actin polymerization in actin wave in stimulated mast cells suggest that the ratio of these nucleators can be readily tuned in a physiological context. Because of the challenges in visualizing their dynamics in unperturbed physiological context, the sequence of events leading to the interplay of these two types of nucleators in living cells remains unclear, and a number of different scenarios have been proposed. For instance, the leading edge of migrating cells involves the formation of Arp2/3-complex-dependent lamellipodia and formin-nucleated filopodia composed of linear actin filaments. There, formin was proposed to act downstream of Arp2/3 by extending some barbed ends in the Arp2/3 nucleated dendritic tree and continues to elongate them.^84^ Consistent with this convergent elongation model, in a cell-free reconstitution system of filopodia formation, diaphanous-related formin mDia2 was found to be recruited after Arp2/3 and actin.^85^ *In vitro*, formin mDia1 was recruited to Arp2/3 nucleated filaments and activated via a rocket-launching mechanism.^86,87^ Conversely, formin has also been proposed to act upstream of Arp2/3 by providing the mother filaments where Arp2/3 can bind and generate new branches.^88,89^

Our characterization of actin wave nucleators confirms the extensive crosstalk between these nucleators. The nucleators are recruited on the membrane and nucleate actin wave with a constant time delay (FMNL1 precedes Arp3 by approximately 2.3 ± 0.7 s, while FMNL1 precedes LifeAct by approximately 4.1 ± 1.29 s). Their mutual dependence and cooperation are also supported by the fact that without any external perturbation, these nucleators experience the spontaneous fluctuation of oscillation amplitudes in a similar trend. In a cryo-electron tomograph of actin waves in *Dictyostelium* cells, the presence of branched filaments and Arp2/3 at the junction was directly visualized.^9^ It was proposed that actin polymerization was initiated at the membrane by VASP and formins, followed by Arp2/3 complex accumulation and branch nucleation. This upstream role of formin relative to Arp2/3 is consistent with our observation that both FMNL1 and mDia3 are recruited 2–4 s before Arp2/3. In addition, because the CA mutant of FMNL1 did not interfere with Arp2/3 recruitment, but a short CA mutant lacking the actin binding FH1-FH2 domains (mini-FMNL1-V281E) negatively regulates Arp2/3 participation, it appears that Arp2/3 indeed benefited from the interaction of FMNL1 with actin, directly or indirectly. However, Arp2/3 is localized as a distinct cluster of puncta. FMNL1 and mDia3 are enriched in the waves but did not display contrasted clusters that colocalize with Arp2/3. FMNL1 appears to be mutually exclusive compared to that of Arp2/3 (Figure 6F). In the cryo-electron tomograph, the densities of these junction and branched filaments were also relatively low compared to the densities of total filaments. Thus, it seems likely that the majority of formin-nucleated filaments remains unbranched in their lifetime. Thus, the synergy most likely takes place at the level of actin networks with spatial proximity, not necessarily at the level of single filaments or branches.

### Competition between Arp2/3 and formins

The regulation of Arp2/3 and formins also competes with each other at multiple time scales. When Arp2/3 is transiently inhibited, the surface recruitment of FMNL1 and mDia3 instantly increased. This antagonism occurs within seconds. There is also a longer timescale competition, because when the CA mutant of formin or even formin was expressed, the recruitment of Arp2/3 was reduced or became undetectable. Upregulation of formin activity upon Arp2/3 inhibition was previously observed in many contexts, including in T cells.^90^ The most prominent model is that Arp2/3 and formin compete for a limited pool of actin monomers.^91–96^ However, our data do not align with this model. First, the limited pool of actin monomers can only explain a competition between Arp2/3 and formin activity, but not their localization and amplitude of recruitment. Second, we consistently observed coordinated amplitudes of FMNL1 and Arp3 oscillations (Figure 6A). This observation suggests that it is possible to increase both Arp3 or FMNL1 recruitments from one cycle of 20 s to the next 20 s for a prolonged duration. If recruitment of one nucleator is competing with another one, then one would expect to see opposite amplitudes for formin and Arp2/3 oscillations. These similar trends in the oscillations of both nucleators over many cycles indicate that the limiting factor dictating the competition, as reflected in the acute Arp2/3 inhibitor experiment, must be short-lived and does not carry over to the next cycle. We therefore favor a model where the limiting factor dynamically resets every cycle (20–30 s). Our results are consistent with active Cdc42 being such a limiting factor. Cdc42-dependent competition between Arp2/3 and formin operates through at least two mechanisms. First, the interactions between FMNL1 and Cdc42 are mutually exclusive from those of WASP and Cdc42. Second, Arp2/3 limits the amount of active Cdc42 through the recruitment of SHIP1, forming a negative feedback from Arp2/3 to PI(3,4,5)P_3_. Previously, we have shown that ventral actin waves in mast cells are coupled with synchronized cycles of clathrin-mediated endocytosis.^30^ It is also possible that the Arp2/3 complex, together with endocytic fission events, limits the dwell time of active Cdc42 on the plasma membrane. The inhibitory role of Arp2/3 on Cdc42 has been overlooked. The dynamic nature of the actin wave therefore makes it an excellent model to dissect the interplay of actin regulators in the context of higher-order assembly. A comprehensive understanding of the intricate processes governing actin nucleation and assembly within these waves holds significant implications for unraveling how their organization and dynamics are matched with function in varied contexts.^3,79,97^

### Limitations of the study

One of the limitations of the study is the spatial resolution of light microscopy. It will be important to characterize the nucleators with a resolution capable of resolving single actin filaments, such as that provided by cryo-electron tomography. Another limitation is that the model involving a limited upstream regulator (e.g., active Cdc42) needs to be tested directly. This may be achieved using methods such as overexpression of guanine nucleotide exchange factors or optogenetic activation of Cdc42.

## STAR★METHODS

### RESOURCE AVAILABILITY

#### Lead contact

Further information and requests for resources and reagents should be directed to and will be fulfilled by the lead contact, Min Wu (wu.min@yale.edu).

#### Materials availability

All unique/stable reagents generated in this study are available from the lead contact without restriction.

#### Data and code availability

All data reported in this paper will be shared by the lead contact upon request. The code used to generate the figures has been previously documented and is openly accessible on GitHub, publicly available (https://github.com/min-wu-lab/2024-Chua-et-al, https://doi.org/10.5281/zenodo.8083400). Source data used for analysis in this paper is uploaded to Mendeley data (https://doi.org/10.17632/kt34mc26k3.1). Any additional information required to re-analyze the data reported in this paper is available from the lead contact upon request.

### EXPERIMENTAL MODEL AND STUDY PARTICIPANT DETAILS

#### Cell culture

Rat Basophilic Leukemia (RBL-2H3) cells (ATCC, CRL-2256) were maintained in monolayer cultures with MEM growth medium (Life Technologies, Carlsbad, CA) supplemented 20% heat inactivated Fetal Bovine Serum (Sigma-Aldrich, St Louis, MO) and harvested with TrypLE Express (Invitrogen). The maximum number of cell passages for cells used in our experiments was limited to 30.

### METHOD DETAILS

#### Cell transfection

For transient transfections, 1.5 × 10^6^ cells were re-suspended in 10 μL of R buffer provided by the transfection kit with 1 μg of plasmid each, followed by two pulses at 1200 mV for 20 ms using Neon Transfection Electroporator (Life Technologies, Carlsbad, CA). After transfection, cells were plated at subconfluent densities in 35 mm glass-bottom Petri dishes (MatTek P35G-1.5-20-C). Transfected cells were maintained at a 37°C humidified incubator overnight.

#### Cell stimulation

For stimulation, transfected cells were sensitized overnight with anti-DNP IgE (Life Technologies, Carlsbad, CA) at 0.5 μg/mL 80 ng/mL of DNP-BSA, a multivalent antigen that stimulates an antigen response, was added 1 h prior to imaging acquisition.

#### Drug treatment

For experiments using inhibitors, they were diluted from stock and added to the cells at the respective final concentrations: CK-666 (50–500 μM, Sigma-Aldrich, St Louis, MO), Cytochalasin-D (2 or 4 μM, Sigma-Aldrich, St Louis, MO). For experiments involving the chronic depletion of F-actin levels, transiently transfected cells were incubated for 22 h in growth medium containing 2–4 μM cytochalasin-D before imaging. For experiments involving the recovery of actin waves in cytochalasin-D treated cells, chronically treated cells can be released into fresh growth medium for 2, 3, or 24 h before imaging.

#### Molecular cloning and plasmids

The GFP-Arp3 (Wu Lab plasmid code A31), constructed by Matt Welch, was a gift from Dorothy Schafer^98^ (Human Arp3 was cloned into pEGFP-N1 using EcoRI and BamH1 sites). mCherry-Arp3 (Wu Lab plasmid code A36) was a gift from Michael Davidson (Addgene plasmid #54981). LifeAct-GFP (Wu Lab plasmid code A11) and LifeAct-mRuby (Wu Lab plasmid code A12) were gifts from Roland Wedlich-Solber (LifeAct was subcloned into pEGFP-N1 using EcoRI and BamHI sites. LifeAct-mRuby was generated by replacing GFP of LifeAct with mRuby.^99^ For both LifeAct constructs, the linker GDPPVAT was generated between LifeAct and GFP or mRuby.^100^ FMNL1-GFP (Wu Lab plasmid code A98) was a gift from Michael Rosen^59^ (FMNL1 was subcloned into a modified pCMV-Script vector containing C-terminal EGFP using BamHI and XhoI sites). GFP- CA-FMNL1 (L1062D mutation) (Wu Lab plasmid code A98F), GFP- FMNL1CT (aa 612–1094) (Wu Lab plasmid code A98C), GFP- mini-FMNL1 (Wu Lab plasmid code A98E), GFP- mini-FMNL1-V281E (Wu Lab plasmid code A98G), GFP- mini-FMNL1-T126D (Wu Lab plasmid code A98H) were gifts from Michael Rosen.^59^ FMNL1-iFP (Wu Lab plasmid code A98K) was generated in our lab and subcloned using XhoI and BamHI sites from FMNL1-GFP where GFP was replaced with iFP fluorescent tag. GFP-mDia3 (Wu Lab plasmid code A99) was constructed by Shuh Narumiya through subcloning the fragment encoding mDia3 from pCR vector into pEGFP vector using AccI and BamHI sites.^101^ mCherry- mDia3 (Wu Lab plasmid code A99C) was generated in our lab and subcloned using SacI and BamHI sites from GFP-mDia3 into mCherry-C1 backbone vector. mCherry- CA-mDia3 (aa 1–1053) (Wu Lab plasmid code A99B) was generated by overlap extension PCR for the deletion of the DAD domain from the full length-mDia3 encoded by mCherry-mDia3. PM-mScarlet (Wu Lab plasmid code M02B) was generated from subcloning of the mScarlet fluorescent tag using PCR from PMScarlet-H C1^102^ into PM-miRFP670 where miRFP670 was replaced with the mScarlet fluorescent tag. Cdc42 BD-GFP (Wu Lab plasmid code G011), a biosensor for detecting endogenous active Cdc42 was generated by cloning bacterial expressing Cdc42 activity sensor pET23-CBD-(-PP)-EGFP^103^ into pEGFP-N1 vector with restriction sites XhoI and BamHI. For experiments using Cdc42 BD-GFP, a stable cell line was generated. Cdc42 BD-GFP plasmid was transfected into RBL-2H3 cells for 24 h, selected in MEM containing 20% FBS and 0.5 mg/mL selection antibiotic G418 and FACS sorted. FBP17-EGFP (Wu Lab plasmid code F011) was subcloned into the pEGFP-N1 vector from EGFP-FBP17 using the BamH1 and HindIII restriction sites. EGFP-FBP17 and GFP-SHIP1 were kind gifts from Pietro De Camilli. To generate GFP-N-WASP (Wu Lab plasmid code A41), the N-WASP sequence was cloned into EGFP-C1 vector using the SacI and EcoRI restriction sites.

#### Microscopy

Before imaging, medium in the dishes were replaced with either pre-warmed fresh normal growth medium, or Tyrode’s imaging buffer containing 20 mM HEPES (pH = 7.4), 135 mM NaCl, 5.0 mM KCl, 1.8 mM CaCl_2_, 1.0 mM MgCl_2_ and 5.6 mM glucose (except for experiments involving chronic cytochalasin-D treatment) and transferred to a heated microscope stage (Live Cell Instrument, Seoul, South Korea) maintained at 37°C throughout the experiments. It is recommended to replace existing medium in dishes with Tyrode’s imaging buffer for acute inhibitor experiments involving CK-666 prior to imaging. To prevent thermal drifting, dishes were allowed to rest on the heated microscope stage for at least 30 min after replacing medium. For TIRFM imaging of live cells’ cortical dynamics, a Nikon Ti-E inverted microscope (Nikon, Shinagawa, Tokyo) was used. The microscope was equipped with a perfect focus system that prevents focus drift, an iLAS2 motorized TIRF illuminator (Roper Scientific, Evry Cedex, France) and either an Evolve 512 EMCCD camera (Photometrics, Tucson, AZ) (16 bit, pixel size 16 μm) or Prime95b sCMOS camera (Photometrics, Tucson, AZ) (16 bit, pixel size 11 μm). All images were acquired using objective lenses from Nikon’s CFI Apochromat TIRF Series (100xH N.A. 1.49 Oil; 60xH N.A. 1.49 Oil). Multi-channel imaging of samples was achieved by the sequential excitation with 491 nm (100 mW), 561 nm (100 mW) and 642 nm (100 mW) lasers, reflected from a quad-bandpass dichroic mirror (Di01- R405/488/561/635, Semrock, Rochester, NY) located on a Ludl emission filter wheel (Carl Zeiss AG, Oberkochen, Germany). The microscope was controlled by a MetaMorph software (Version 7.8.6.0) (Molecular Devices, LLC, Suunyvale, CA). For sequential TIRF/Differential interference contrast (DIC) imaging, channel transition was controlled by Multiple Dimension Acquisition module in Metamorph and a Normaski primary prism was inserted in condenser. During image acquisition, the samples were maintained at 37°C with an on-stage incubator system (Live Cell Instrument, Seoul, South Korea). To ensure cell viability during long term imaging lasting more than 1 h, spent media was replaced with fresh media. In addition, 5% humidified CO_2_ was supplied. All image sequences were acquired at time intervals of 0.6–3 s, except for extended acquisition durations lasting over 45 min, which were acquired at time intervals of 4 s.

#### Image analysis

Post-acquisition image analyses were performed by either by Fiji^104^ or MATLAB (The MathWorks, Inc., Natick, MA). Kymographs were generated using the ‘Reslice’ tool. Depending on intensity of the fluorescent markers, ‘average’ projection filters (average of 10 frames) and background subtractions may be applied to enhance presentation. For consistency, the same image processing was applied across all channels for kymographs generated from multi-color imaging and applied identically across different conditions that were directly compared to each other. Sequential montages depicting the wave front or punctum tracking were generated using the ‘Make Montage’ tool. For single punctum tracking, a region of interest with a 15 × 15 pixels size was used for all movies. For intensity profiles, a region of interest with a 20 × 20 pixels size was used for all movies. The intensity data points generated by Fiji were normalized and plotted by MATLAB. To illustrate phase differences between proteins in wave propagation, multiple cycles of their intensity fluctuations were aligned. The solid lines represent the mean intensities and shaded region represent the standard deviations of the intensities. To obtain the relative intensity, the intensity values were first background subtracted using a region outside of the cell. It was then normalized by the background intensity of the cell defined as the average of the lowest 50 points in the intensity profile). If the normalization was correct, baseline of the oscillation should be around 1, and the peak intensity was used as an indicator for oscillation amplitude. Peaks in the timeseries were defined as local maximal (intensity higher than all its neighbors within +/− 10 s). Cells positive of regular oscillation was determined by visual impression of kymographs and confirmed using the built-in Fast Fourier Transform function in MATLAB. Cells that gave an output of a single FFT peak within the designated range (typically 10–100 s for Cdc42 oscillation) were deemed positive and cells without the FFT peak were deemed negative of waves. Wavelet analysis, autocorrelation, cross-correlation, phase plots, interpeak interval analysis and peak amplitude analysis were performed using MATLAB. Raincloud plot and boxplot were generated with open source codes developed previously.^105^ Custom codes used for analysis are deposited on Github (https://github.com/min-wu-lab/2024-Chua-et-al, https://doi.org/10.5281/zenodo.8083400) and is publicly available.

### QUANTIFICATION AND STATISTICAL ANALYSIS

All statistical analysis were performed by Prism 10 (GraphPad Software, Inc, La Jolla, CA). For comparisons of oscillatory periodicities exhibited among untreated and cytochalasin-D-treated cells of different recovery durations in Figure 3C, one-way ANOVA, followed by Dunnett’s multiple comparison tests were performed to compare the mean for each dataset against the untreated (control) dataset. For comparisons of peak amplitudes and IPI analysis among samples in Figures 4I–4K, one-way ANOVA, followed by Dunnett’s multiple comparison tests were performed to compare the mean for each dataset against a control dataset. For comparison of peak amplitudes and IPI analysis between control (untreated) and samples acutely treated with CK-666 in Figure 7D and S7, unpaired two-tailed student’s t-test was performed. Unless otherwise stated, error bars for all data shown represents mean ± S.E.M.

## Supplementary Material

1

2

3

5

6

7

8

9

10

11

12

## Figures and Tables

**Table:** KEY RESOURCES TABLE

REAGENT or RESOURCE	SOURCE	IDENTIFIER

Chemicals, peptides, and recombinant proteins		

CK-666	Sigma-Aldrich	SML0006
Cytochalasin-D	Sigma-Aldrich	C8273
FBS	Millipore Sigma	F4135
MEM	Life Technologies	11095098
TrypLE Express	Invitrogen	12604021
Puromycin	Gibco	A1113803

Experimental models: Cell lines		

RBL-2H3 cell	ATCC	Cat#: CRL-2256; RRID:CVCL_0591
RBL-2H3 cell stably expressing Cdc42 BD-GFP	–	Wu Lab Cell Line ID: B-ae

Recombinant DNA		

GFP-Arp3	Gift from Dorothy Schafer	Wu Lab Plasmid ID: A31
mCherry-Arp3	Gift from Michael Davidson (Addgene #54981)	Wu Lab Plasmid ID: A36
LifeAct-GFP	Gift from Roland Wedlich-Solber	Wu Lab Plasmid ID: A11
LifeAct-mRuby	Subcloned by replacing GFP of LifeAct-GFP with mRuby tag	Wu Lab Plasmid ID: A12
FMNL1-GFP	Gift from Michael Rosen	Wu Lab Plasmid ID: A98
GFP-CA-FMNL1	Gift from Michael Rosen	Wu Lab Plasmid ID: A98i
GFP-FMNL1CT	Gift from Michael Rosen	Wu Lab Plasmid ID: A98c
GFP-mini-FMNL1-T126D (inactive)	Gift from Michael Rosen	Wu Lab Plasmid ID: A98g
GFP-mini-FMNL1-V281E (active)	Gift from Michael Rosen	Wu Lab Plasmid ID: A98h
GFP-mDia3	Gift from Shuh Narumiya	Wu Lab Plasmid ID: A99
mCherry-mDia3	Subcloned from GFP-mDia3, gift from Shuh Narumiya	Wu Lab Plasmid ID: A99c
mCherry-CA-mDia3	Subcloned from mCherry-mDia3 with overlap extension PCR	Wu Lab Plasmid ID: A99b
PM-mScarlet	Subcloned from PM-miRFP670 with PCR to replace fluorescent tag	Wu Lab Plasmid ID: M02b
Cdc42 BD-GFP	Subcloned into pEGFP-N1 vector from pET23-CBD-(-PP)-EGFP	Wu Lab Plasmid ID: G011
FBP17-EGFP	Gift from Pietro De Camilli	Wu Lab Plasmid ID: F011
GFP-SHIP1	Gift from Pietro De Camilli	Wu Lab Plasmid ID: P87
GFP-N-WASP	Subcloned into EGFP-C1 vector	Wu Lab Plasmid ID: A41

Software and algorithms		

Custom codes for analysis	Written in-house	https://github.com/min-wu-lab/2024-Chua-et-al, https://doi.org/10.5281/zenodo.8083400
Fiji (ImageJ)	NIH	http://fiji.sc/
Prism 10	Graphpad	N/A
MATLAB	Mathworks	N/A

Other		

Neon Transfection System 10 μL Kit	Life Technologies	MPK1096
35 mm Dish | No. 1.5 Coverslip | 20 mm Glass Diameter | Uncoated	Mattek Corporation	P35G-1.5-20-C

## Highlights

- Formins (FMNL1 and mDia3) are recruited to cortical actin waves prior to Arp2/3
- Waves of Cdc42 and active FMNL1 can occur in the absence of actin waves
- FMNL1 and Arp2/3 compete with each other for access to active Cdc42
- Arp2/3 clusters SHIP1 and negatively regulates Cdc42
