# Supplementary material for: Competition and synergy of Arp2/3 and formins in nucleating actin waves
Source: Cell Rep. Author manuscript; Available in PMC 2024 Sep 6. (PMC11378572; doi:10.1016/j.celrep.2024.114423)
Supplement: 1 [file NIHMS2011875-supplement-1.pdf]

Cell Reports, Volume *43*

Supplemental information

Competition and synergy of Arp2/3

and formins in nucleating actin waves

Xiang Le Chua, Chee San Tong, Maohan Su, X.J. Xǔ, Shengping Xiao, Xudong Wu, and Min Wu

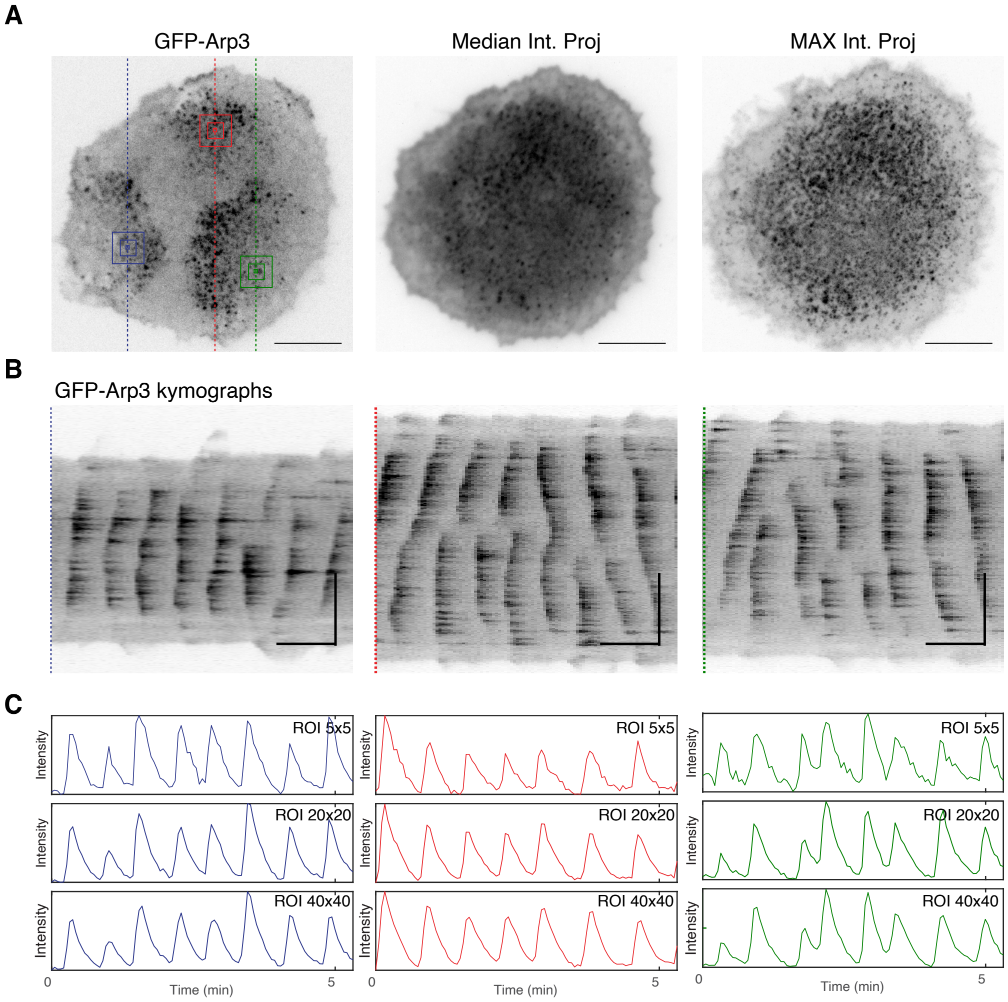

Figure S1. Clusters of Arp2/3 puncta assemble in waves concentrated in the middle of cell.

(A) Representative micrographs of a cell expressing GFP-Arp3. Median- and maximum intensity projections of timelapse images of the representative cell exhibiting waves of GFP-Arp3 puncta. (B) Kymographs re-sliced from 10 continuous frames with average projection from three arbitrarily chosen region of the cell (left: blue; middle: red; right: green) to demonstrate cycles of wave propagation over time. (C) Fluorescence intensity plots derived from three arbitrarily chosen regions of interests (ROIs) of different pixel sizes (5 x 5 pixels; 20 x 20 pixels; 40 x 40 pixels).

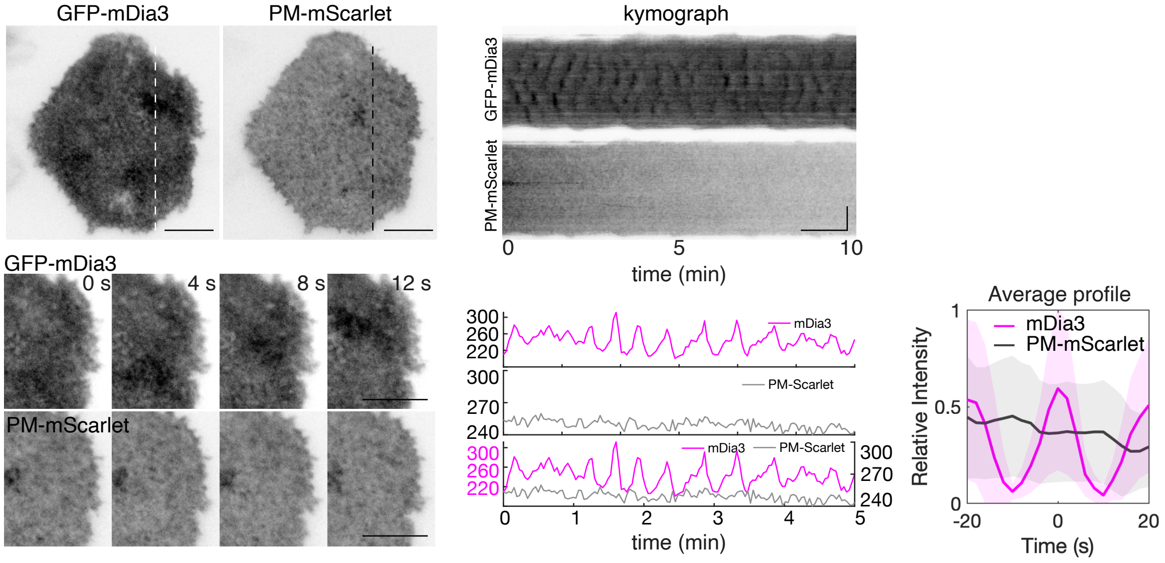

Figure S2. Cortical waves of GFP-mDia3 compared with a plasma membrane marker.

Representative micrographs and kymographs of a cell co-expressing GFP-mDia3 and PM-mScarlet. Representative intensity plots of a cell co-expressing GFP-mDia3 and PM-mScarlet. Representative average intensity profile of PM-mScarlet aligned with respect to GFP-mDia3. Horizontal scale bars in micrographs: 10 μm. Vertical scale bars in kymographs: 10 μm. Horizontal scale bars in kymographs: 1 min.

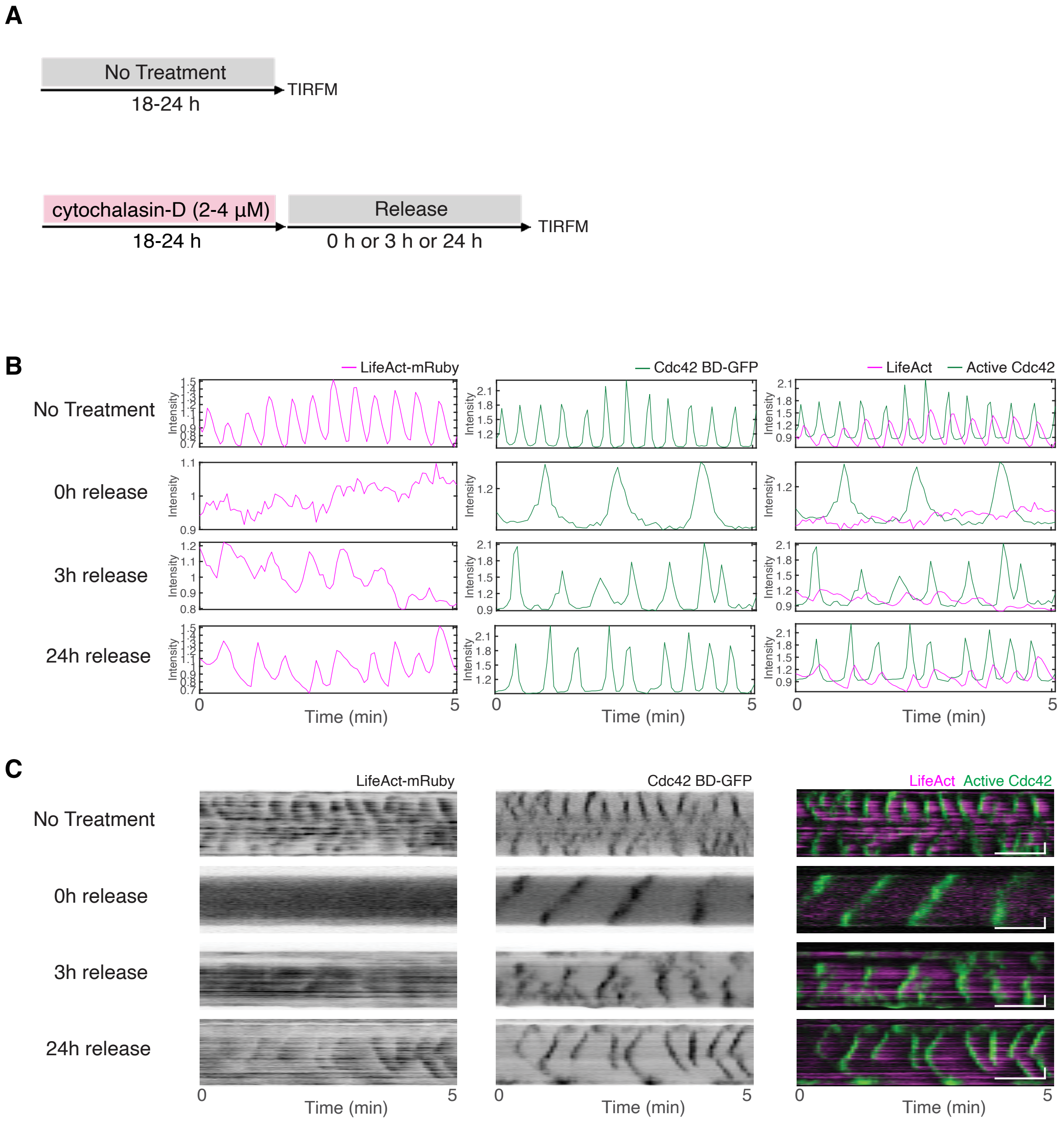

Figure S3. Oscillations of active Cdc42 relative to the recovery of F-actin waves.

(A) Treatment schematics for cells under (top) untreated conditions or (bottom) depleted of F-actin with cytochalasin-D with different release time. (B-C) Representative intensity plots (B) and kymographs (C) of untreated and cytochalasin-D-treated cells stably-expressing Cdc42 BD-GFP co-transfected with LifeAct-mRuby with different release durations. Vertical scale bars in kymographs: 10 µm. Horizontal scale bars in kymographs: 1 min. Cytochalasin-D inhibitor experiments were performed in normal growth medium.

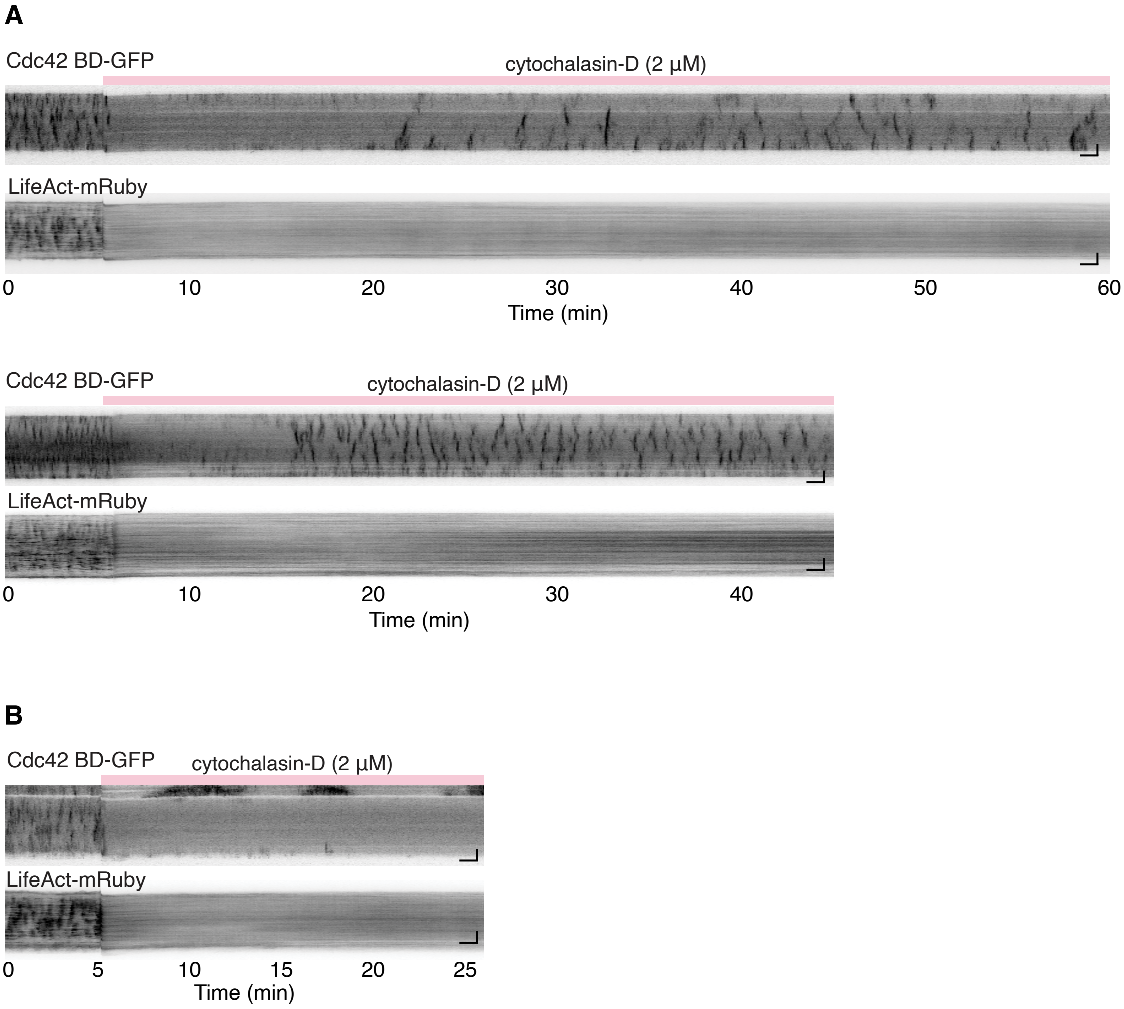

Figure S4. Cortical traveling waves of active Cdc42 and LifeAct before and after treatment with cytochalasin-D

(A-B) Kymographs of cells stably-expressing Cdc42 BD-GFP co-transfected with LifeAct-mRuby before and after treated with cytochalasin-D (2 μM). Cytochalasin-D inhibitor experiments were performed in normal growth medium.

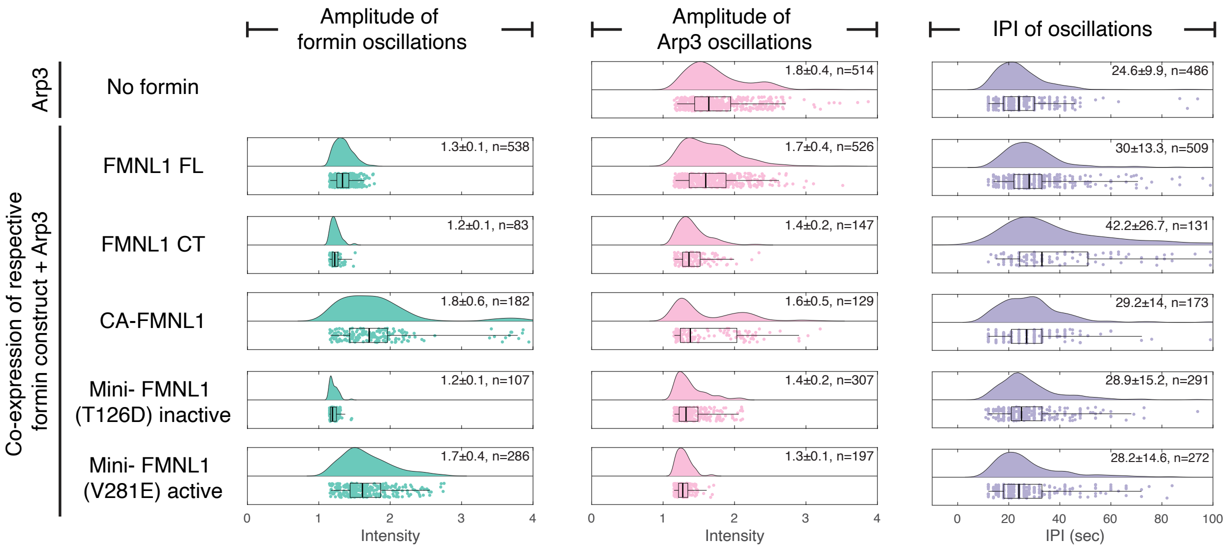

Figure S5. Quantifications for the distribution of average peak amplitudes and IPI exhibited by the co-expressions of Arp3 with FMNL1 or FMNL1 mutants.

Analyses of peak amplitudes (green and pink columns) and IPI (purple column) of cells either expressing mCherry-Arp3, or co-expressing mCherry-Arp3 with different formin constructs. The raincloud plots combining data distribution (the ‘cloud’), jittered raw data (the ‘rain’) and box-and-whisker plots (box: 1st/3rd quartiles; whisker: 2nd and the 98th percentile) are shown. n indicates the number of peaks or interpeak intervals.

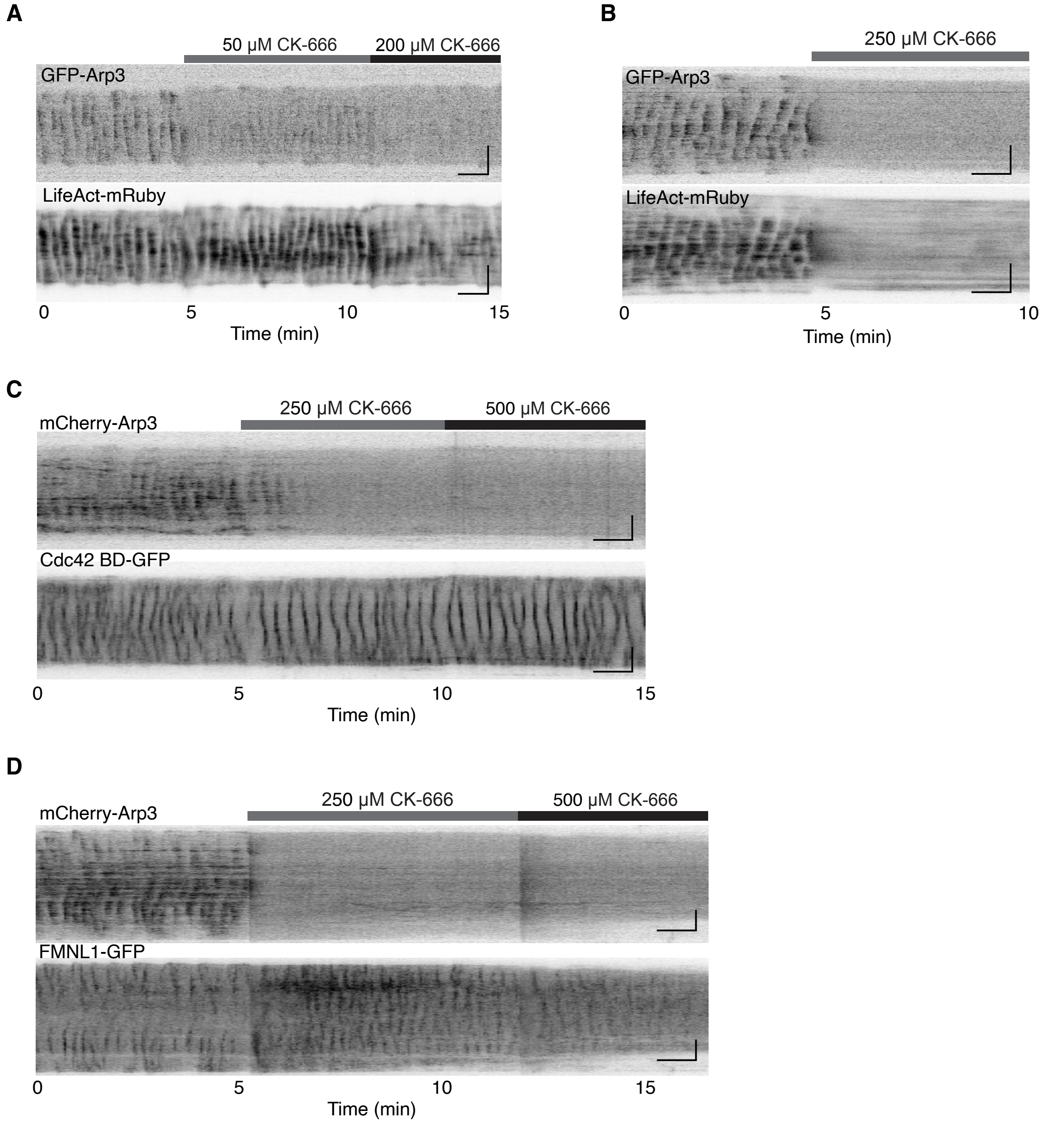

Figure S6. Cortical waves of active Cdc42, actin nucleators and F-actin in response to high dosage of CK-666.

(A) Representative of kymograph of a cell co-expressing GFP-Arp3 and LifeAct-mRuby before and after treated with 250 μM of CK-666. (B) Representative of a cell co-expressing GFP-Arp3 and LifeAct-mRuby before and after treated with 50 μM, followed by 200 μM CK-666. (C-D) Representative of a cell co-expressing mCherry-Arp3 and (C) Cdc42 BD-GFP or (D) FMNL1-GFP, before and after treated with 250 μM, followed by 500 μM CK-666. Vertical scale bars: 10 μm. Horizontal scale bars: 1 min. CK-666 inhibitor experiments were performed in Tyrode's imaging buffer.

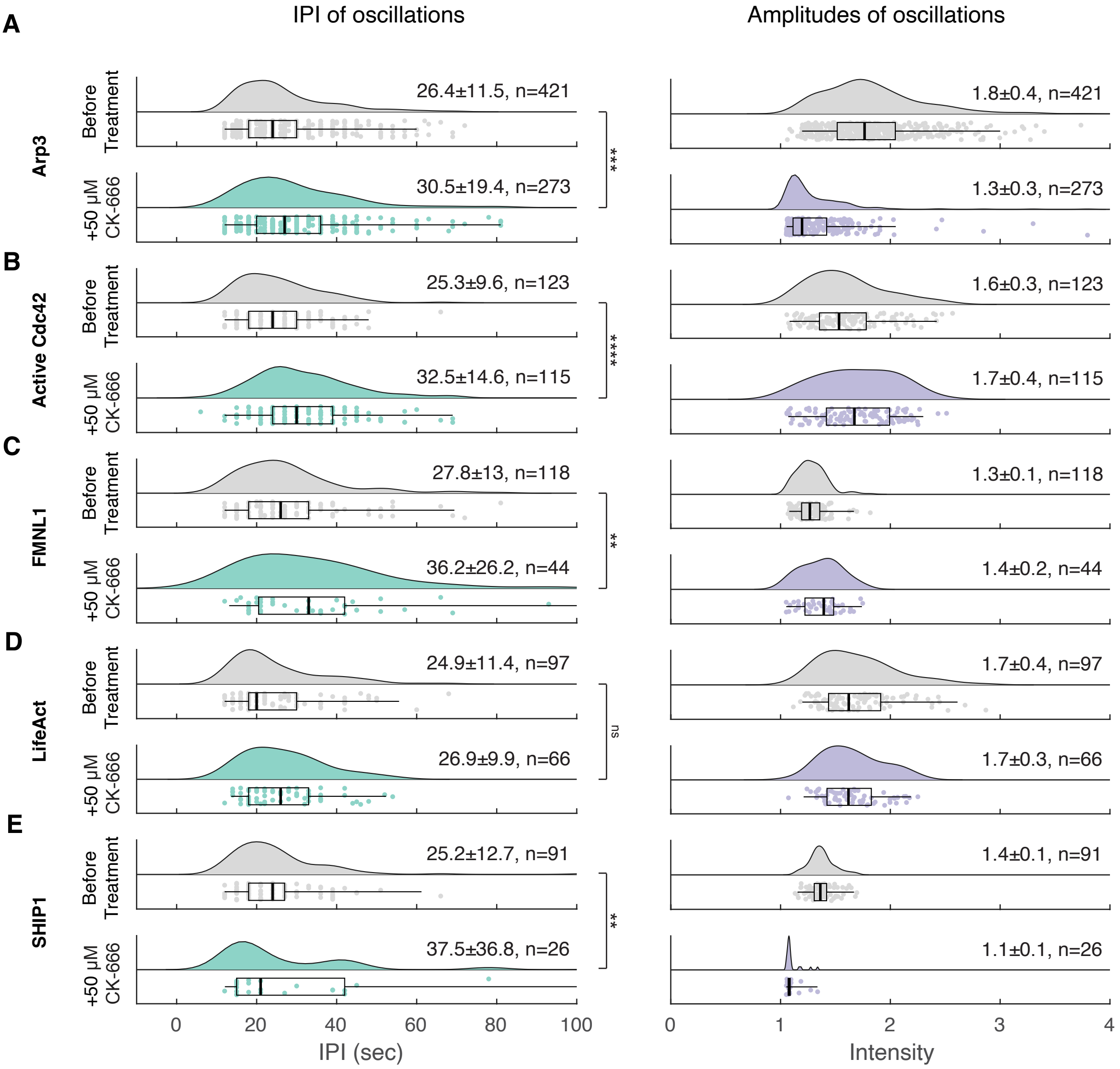

Figure S7. Quantifications for the IPI and peak amplitudes before and after treatment by CK-666.

Average IPI (Left column) and peak amplitude (Right column) exhibited by cells expressing (A) GFP-Arp3 or mCherry-Arp3, (B) Cdc42 BD-GFP, (C) FMNL1-GFP, (D) LifeAct-mRuby, or (E) GFP-SHIP1, before (grey) and after treatment by 50 µM CK-666 (green). The raincloud plots combining data distribution (the 'cloud'), jittered raw data (the 'rain') and box-and-whisker plots (box: 1st/3rd quartiles; whisker: 2nd and the 98th percentile) are shown. n indicated the number of peaks. Statistical significance: ****p < 0.0001; ***p < 0.0006; **p = 0.0072; ns = not significant, t-test. CK-666 inhibitor experiments were performed in Tyrode's imaging buffer.
